# Apatinib triggers autophagic and apoptotic cell death via VEGFR2/STAT3/PD-L1 and ROS/Nrf2/p62 signaling in lung cancer

**DOI:** 10.1186/s13046-021-02069-4

**Published:** 2021-08-24

**Authors:** Chunfeng Xie, Xu Zhou, Chunhua Liang, Xiaoting Li, Miaomiao Ge, Yue Chen, Juan Yin, Jianyun Zhu, Caiyun Zhong

**Affiliations:** 1 Department of Nutrition and Food Safety, School of Public Health, Nanjing Medical University, 101 Longmian Ave, Jiangning, Nanjing, 211166 China; 2 Department of Laboratory, The Affiliated Suzhou Hospital of Nanjing Medical University, Suzhou Municipal Hospital, Gusu School, Nanjing Medical University, 242 Guangji Rd, Suzhou, 215008 China; 3 Cancer Research Division, Center for Global Health, School of Public Health, Nanjing Medical University, Nanjing, 211166 Jiangsu China

**Keywords:** NSCLC, Apatinib, Apoptosis, Autophagy, STAT3/PD-L1, ROS/Nrf2/p62

## Abstract

**Background:**

Recently, a variety of clinical trials have shown that apatinib, a small-molecule anti-angiogenic drug, exerts promising inhibitory effects on multiple solid tumors, including non-small cell lung cancer (NSCLC). However, the underlying molecular mechanism of apatinib on NSCLC remains unclear.

**Methods:**

MTT, EdU, AO/EB staining, TUNEL staining, flow cytometry, colony formation assays were performed to investigate the effects of apatinib on cell proliferation, cell cycle distribution, apoptosis and cancer stem like properties. Wound healing and transwell assays were conducted to explore the role of apatinib on migration and invasion. The regulation of apatinib on VEGFR2/STAT3/PD-L1 and ROS/Nrf2/p62 signaling were detected. Furthermore, we collected conditioned medium (CM) from A549 and H1299 cells to stimulate phorbol myristate acetate (PMA)-activated THP-1 cells, and examined the effect of apatinib on PD-L1 expression in macrophages. The Jurkat T cells and NSCLC cells co-culture model was used to assess the effect of apatinib on T cells activation. Subcutaneous tumor formation models were established to evaluate the effects of apatinib in vivo. Histochemical, immunohistochemical staining and ELISA assay were used to examine the levels of signaling molecules in tumors.

**Results:**

We showed that apatinib inhibited cell proliferation and promoted apoptosis in NSCLC cells in vitro. Apatinib induced cell cycle arrest at G1 phase and suppressed the expression of Cyclin D1 and CDK4. Moreover, apatinib upregulated Cleaved Caspase 3, Cleaved Caspase 9 and Bax, and downregulated Bcl-2 in NSCLC cells. The colony formation ability and the number of CD133 positive cells were significantly decreased by apatinib, suggesting that apatinib inhibited the malignant and stem-like features of NSCLC cells. Mechanistically, apatinib inhibited PD-L1 and c-Myc expression by targeting VEGFR2/STAT3 signaling. Apatinib also inhibited PD-L1 expression in THP-1 derived macrophages stimulated by CM from NSCLC cells. Furthermore, apatinib pretreatment increased CD69 expression and IFN-γ secretion in stimulated Jurkat T cells co-cultured with NSCLC cells. Apatinib also promoted ROS production and inhibited Nrf2 and p62 expression, leading to the autophagic and apoptotic cell death in NSCLC. Moreover, apatinib significantly inhibited tumor growth in vivo.

**Conclusion:**

Our data indicated that apatinib induced autophagy and apoptosis in NSCLC via regulating VEGFR2/STAT3/PD-L1 and ROS/Nrf2/p62 signaling.

**Supplementary Information:**

The online version contains supplementary material available at 10.1186/s13046-021-02069-4.

## Highlights

1. Apatinib suppressed proliferation, induced cell cycle arrest and apoptosis, and inhibited malignancy in NSCLC in vitro and in vivo.
2. Apatinib downregulated PD-L1 and c-Myc in NSCLC through VEGFR2/STAT3 pathway.
3. Apatinib inhibited PD-L1 expression in THP-1 derived macrophages stimulated by the conditioned medium from NSCLC cells and partially restored the activation of Jurkat T cells co-cultured with NSCLC cells.
4. Apatinib induced ROS generation and inhibited Nrf2 and p62 expression, leading to the autophagic and apoptotic cell death in NSCLC.

**Supplementary Information:**

The online version contains supplementary material available at 10.1186/s13046-021-02069-4.

## Background

Lung cancer is the leading cause of cancer-related deaths worldwide, with a very low 5-year survival rate (less than 15%) for all stage combined [1, 2]. Non-small cell lung cancer (NSCLC) is the most common histologic subtype, accounting for approximately 85% of all lung cancer cases [3, 4]. About half of the NSCLC patients present with an advanced stage at their first diagnosis, causing the subsequent therapy failure. Moreover, the outcome of locally advanced NSCLC patients remains poor, because of the almost inevitable metastasis, chemoresistance and subsequently tumor relapse [5, 6]. Thus, alternatives to conventional treatment are urgently warranted for NSCLC.

NSCLC is a highly vascularized tumor and inhibition of angiogenesis has become a promising therapeutic approach [7]. It is reported that vascular endothelial growth factor (VEGF) plays an important role in the angiogenic signaling and vascular endothelial growth factor receptors (VEGFRs) are tyrosine kinases that serve as key regulators of this process. VEGFR2 is expressed by tumor cells and targeting this receptor may simultaneously affect tumor stroma (i.e. blood vessels) and tumor parenchyma, as it has been demonstrated in a variety of tumor types, including NSCLC [8]. Apatinib is a novel tyrosine kinase inhibitor (TKI) that approved and launched in China with promising therapeutic efficacy and tolerance in the treatment of multiple solid tumors [9]. Apatinib exerts its antigenic effects by inhibiting VEGFR-induced proliferation and migration of endothelial cells via highly and selectively targeting VEGFR2 [10]. Multiple clinical trials have demonstrated the antitumor activity of apatinib in monotherapy or combination therapy in advanced NSCLC patients [11–13]. Considering numerous molecules and signaling pathways influence the angiogenic response, in-depth understanding of the inhibition role of apatinib on NSCLC and its underlying mechanism are urgently needed for the further clinical usages.

Accumulating evidence have reported that antiangiogenic agents decrease the immunosuppression by modulating tumor microenvironment (TME) and exhibit synergetic effects when combined with immunotherapies [14]. Previous studies have shown that apatinib exerts its immunomodulatory activity in several tumor types. Zhao et al. reported that low-dose apatinib optimized the immunosuppressive TME and enhanced the immune response in murine lung carcinoma models and in patients with advanced nonsquamous NSCLC in a phase III trial [15]. Wang et al. showed that apatinib overcame the innate resistance to programmed death 1(PD-1)/programmed death ligand 1 (PD-L1) blockade and significantly improved the antitumoral activity of PD-1 antibody in advanced triple-negative breast cancer [16]. Further studies reported that apatinib inhibited PD-L1 expression by targeting signal transducer and activator of transcription 3 (STAT3) in osteosarcoma [17], while promoted its expression to enhance anti-PD-1 therapy in colon cancer [18]. In addition, apatinib improved the efficacy of immunotherapy by preventing the dysfunction of natural killer cells in hepatocellular carcinoma [19]. However, whether apatinib could affect PD-L1 and its regulatory mechanism in NSCLC remains unclear.

The nuclear factor erythroid-derived 2-like 2 (Nrf2) serves as a key transcriptional activator and its induction is of importance for protecting cells against oxidative and xenobiotic damage. Previous studies have demonstrated that Nrf2 disrupts oxidative stress mediated-cell death via scavenging cellular reactive oxygen species (ROS) and restoring redox balance. Moreover, abnormal activation of Nrf2 is closely associated with development and chemoresistance of a variety of human cancers, especially NSCLC [20]. Therefore, targeting Nrf2 and inducing ROS-mediated cell death might be a promising strategy in cancer treatment. Accumulating evidence have illustrated that Nrf2 also plays a vital role in autophagy regulation by forming a positive feedback with SQSTM1/p62, an autophagy adaptor protein. It is reported that insufficient autophagy causes p62 accumulation, which further sequesters kelch-like ECH-associated protein 1 (Keap1), the negative regulator of Nrf2, and leads to the stabilization of Nrf2 [21]. Thus, promoting autophagy and breaking the crosstalk between p62 and Nrf2 might be a potential option for cancer chemoprevention [22, 23]. Sun et al. showed that apatinib promoted ROS-dependent apoptosis and autophagy by negatively modulating Nrf2 and p62 in ovarian cancer cells [24]. Other studies also reported that apatinib inhibited cell growth and metastasis and promoted apoptosis by regulating autophagy in a variety of human cancers [25, 26]. However, whether apatinib could inhibit NSCLC by directly promoting autophagy and interfering with Nrf2 and p62 remains unclear.

Therefore, our present study aimed to explore the underlying mechanism of the inhibitory effects of apatinib on NSCLC. We showed that apatinib induced cell death of lung cancer by inhibiting cell proliferation, causing cell cycle arrest, and inducing apoptosis and autophagy. We also illustrated that apatinib suppressed malignant phenotype by inhibiting migration, invasion, and cancer stem cell-like properties. Mechanistically, apatinib decreased the expressions of PD-L1 and c-Myc by targeting VEGFR2/STAT3 pathway. In addition, apatinib induced autophagy and apoptosis by regulating ROS/Nrf2/p62 signaling. Hence, our study proposed the possible mechanism of apatinib against NSCLC and demonstrated the value of apatinib as a promising therapeutic drug for NSCLC.

## Materials and methods

### Cell lines and cell culture

Human NSCLC cell lines A549 and H1299, monocyte cell line THP-1 and Jurkat T cells were obtained from the Cell Bank of Chinese Academy of Sciences (Shanghai, China). The above cells were cultured in RPMI-1640 (Gibco, USA) with 10% fetal bovine serum (FBS) and 1% antibiotics.

To analyze the effect of apatinib on NSCLC, A549 and H1299 cells were treated with different concentrations of apatinib (0, 2, 5, 10 μM), using 0.1% dimethyl sulfoxide (DMSO) as the solvent control and cell morphology was observed under an inverted microscope ((Nikon, Tokyo, Japan). For combination treatment, cells were exposed to apatinib with or without 5 mM of N-acetyl-L-cysteine (NAC, Sigma-Aldrich, St. Louis, USA) or 20 ng/mL of IL-6 (Peprotech, Rocky Hill, NJ, USA). Apatinb were obtained from Hengrui Medicine Co. Ltd. (Jiangsu, China) and dissolved in DMSO (Sigma-Aldrich).

### 3-(4, 5-Dimethyl-2-thizolyl)-2, 5 diphenyltertazolium bromide (MTT) assay

The effect of apatinib on cell viability was measured by MTT assay (Sigma-Aldrich). Briefly, A549 and H1299 cells (2000 cells/well) were cultured in 96-well plates and exposed to apatinib at indicated concentrations (0, 0.5, 1, 2, 5, 10, 20, 40 μM) for 24 h or 48 h. After incubation with 20 μL of MTT solution (5 mg/ml in PBS) for an additional 4 h at 37 °C, the supernatant solution was removed, 150 μL of DMSO was added to dissolve the formazan crystal, and the absorbance of each well was measured with a microtiter reader (Tecan Infinite 200, Switzerland) at a wavelength of 490 nm. The absorbance values for control cells were set as 1 for normalization.

### 5-Ethynyl-2′-Deoxyuridine (EdU) assay

The EdU assay was conducted using EdU Cell Proliferation Kit with Alexa Fluor488 (Beyotime, Shanghai, China) according to the manufacturer’s protocol. Briefly, A549 and H1299 cells were plated onto the 12-well plates and incubated with apatinib (0, 2, 5, 10 μM) for 48 h. After incubation of 10 μM EdU for 2 h, cells were fixed with 4% paraformaldehyde for 10 min and permeabilized with 0.3% Triton X-100 for 20 min. Then, the incorporated EdU was visualized by means of a click reaction using Alexa Fluor 488 azide for 30 min and the nuclear DNA was stained with 4, 6-diamino-2-phenyl indole (DAPI, Beyotime) for 10 min. Finally, the proliferative cells were observed using a fluorescence microscope (Nikon) and the percentage of EdU positive cells was assessed using the ImageJ software.

### Acridine orange/ethidium bromide (AO/EB) staining

A549 and H1299 cells were cultured with apatinib for 24 h, and the AO/EB staining was performed according the manufacturer’s instructions (SenBeiJia Biological Technology, Nanjing, China). Briefly, cells were incubated with AO/EB solution in the dark for 15 min and the morphology of live and dead cells was observed immediately under a fluorescent microscope the percentage of dead cells was assessed using the ImageJ software.

### Terminal deoxynucleotidyl transferase-mediated dUTP Nick end labeling (TUNEL) staining

The TUNEL assay was conducted using Colorimetric TUNEL Apoptosis Assay Kit (Beyotime, Shanghai, China) according to the manufacturer’s protocol. Briefly, after incubation of apatinib for 48 h, A549 and H1299 cells were fixed, permeabilized, and then inactivated endogenous peroxidase by 0.3% H_2_O_2_ for 20 min. Subsequently, the cells were incubated with a mix solution containing enzyme terminal deoxynucleotide transferase (TdT) and biotinylated (Bio-16) dUTP in TdT buffer at 37 °C for 60 min and the nuclei were stained with diaminobenzidine for 10 min and counterstained with hematoxylin. Finally, the apoptosis cells were visualized by an invert microscope.

### Flow cytometry analysis of cell cycle

After treatment of apatinib for 48 h, A549 and H1299 cells were collected, resuspended, and fixed with precooled 70% ethanol at -20 °C overnight. After stained with the propidium iodide (PI) solution (BD, Biosciences, USA) for 15 min, the distribution of cell cycle phase was measured by flow cytometry with a BD FACSArray (BD). A minimum of 20,000 cells were guaranteed before analysis.

### Plate colony formation assay

A549 and H1299 cells were seeded into 60 mm diameter plates at a density of 100 cells/well. Cells were cultured with different concentrations of apatinib and the medium was renewed every 3 days. After 2 weeks’ culturing, the colonies were fixed and dyed with crustal violet (Beyotime) for 30 min. The colony images were recorded using a digital camera and the numbers of colonies (> 50 cells/colony) were counted via Image J software.

### Anchorage-independent growth assay

Soft agar dishes were precoated with 0.7% agarose (Beyotime) in RPMI-1640 medium. A549 and H1299 cells were plated at a density of 5000 cells/well in 0.35% agarose over the agar base and the medium was fleshed every 3 days with various concentrations of apatinib for 2 weeks, colonies with diameters > 50 μm were counted.

### Flow cytometry analysis of CD133 positive cells

The analysis was performed according to as previously described [27]. Briefly, A549 and H1299 cells were cultured in a stem cell specific serum-free medium and treated with apatinib for 7 days. The sphere-forming cells were collected and incubated with APC-conjugated human monoclonal CD133/1 (AC133) antibody at 4 °C for 15 min. The percentage of CD133 positive cells was detected through flow cytometry (BD). Human monoclonal CD133/1 (AC133) antibody was purchased from Miltenyi Biotech (Teterow, Germany).

### Wound healing assay

A549 and H1299 cells were seeded in 6-well plates for adherent culture and a sterile pipette tip was used to make the scratches. The wounded cells were then treated with apatinib (0, 2, 5, 10 μM) after removing debris by PBS for 48 h. The scratch area was captured at 0 h and 48 h and the migration rate were measured by Image J.

### Transwell assays

A549 and H1299 cells were treated with apatinib for 48 h and were resuspended at a density of 5 × 10^4^ cells/ well (200 μL) and seeded in the upper chambers (24-well migration chambers, 8.0 μm pore membrane, Corning, New York, USA) in the serum-free medium. The upper chambers were precoated with Matrigel (Corning) for cell invasion, not for cell migration. Then, the lower chambers were filled with 800 μL of the medium with 10% FBS. After 24 h incubation, the non-migrated and non-invaded cells in the upper chambers were removed by cotton swabs, and the migrated or invaded cells on the undersurface of the chambers were fixed and stained with crystal violet. The images were observed using a microscope and migrated and invaded cells were measured by Image J.

### ROS detection

A549 and H1299 cells (1 × 10^6^ cells/well) were seeded in 6-well plates. After treatment with apatinib for 24 h, cells were incubated with 5 mM of 2′,7′-dichlorodihydrofluorescein diacetate (DCFDA, Sigma-Aldrich) and placed in a shaker at 37 °C for 20 min. The DCF fluorescence intensity was then immediately detected using a fluorescence microscopy.

### Conditioned medium preparation and macrophages treatment

To obtain conditioned medium (CM) of A549 and H1299 cells, both cells, up to 80% confluence, were washed and cultured with fresh medium for additional 24 h. The cell-medium was collected and centrifuged at 3000 rpm for 10 min at 4 °C.

To analyze the effects of apatinib on macrophages, THP-1 cells were stimulated by pretreatment with 100 ng/mL phorbolmyristate acetate (PMA, Sigma-Aldrich) for 48 h. The THP-1 derived macrophages were then washed and cultured with fresh medium or CM from A549 or H1299 cells with or without 10 μM apatinib for another 4 h.

### Co-culture assay

A549 and H1299 cells were seeded onto 24-well plate at a density of 5 × 10^4^ and treated with or without 10 μM apatinib for 24 h, then the culture medium was aspirated and replaced with fresh 1640 medium containing 10% FBS. The Jurkat T cells were pre-activated with 2 μg/ml soluble anti-CD3 (eBioscience, San Diego, CA, USA) and 1 μg/ml soluble anti-CD28 (eBioscience) for 24 h, and were then mixed with A549 and H1299 control cells or apatinib pretreated cells at a density of 5 × 10^5^ for co-culture. After 24 h, the culture medium was collected for IFN-γ ELISA assay and Jurkat cells were collected for flow cytometry analysis.

### Flow cytometry analysis of CD69 expression

Jurkat T cells from the co-culture system were harvested and washed with PBS. After staining with the PE-cy7 conjugated anti-CD69 antibody (Biolegend, San Diego, CA, USA) in PBS for 30 min in the dark, the stained cells were acquired by flow cytometry analysis. Mean fluorescent intensity was calculated.

### Enzyme-linked immunosorbent assay (ELISA) for interferon-γ (IFN-γ)

IFN-γ level in medium of the co-culture system was determined using Human IFN-γ ELISA kit (CUSABIO, Wuhan, China) according to the manufacturer’s instructions. Optical density was measured at 450 nm, and the amount of IFN-γ was calculated from a standard curve prepared using the recombinant protein.

### Quantitative real-time PCR (qRT-PCR) analysis

Total RNA was isolated with TRIzol reagent (Invitrogen, Carlsbad, CA, USA) according to the manufacturer’s protocol and cDNA was completed using a cDNA synthesis kit (Applied Biological Materials, Canada). qRT-PCR were performed using the EvaGreen 2 × qPCR MasterMix (Applied Biological Materials) and detected by a LightCycler96 real-time detection (Roche, Basel, Switzerland). The PCR primer sequences were as follows: *VEGFR2*: forward: 5′-TGGTCAGGCAGCTCACAGTCC-3′, reverse: 5′-GTTCCGGTTCCCATCCTTCAATAC-3′, *PD-L1*: forward: 5′-CATGTCAGGCTGAGGGCTAC-3′, reverse: 5′-TGGAATTGGTGGTGGTGGTC-3′; *GAPDH*: forward: 5′-CAAGGTCACCATGACAACTTTG-3′, reverse: 5′-GTCCACCACCCTGTTGCTGTAG-3′. *GAPDH* was regarded as an internal reference. The relative expression levels were calculated by the 2^−ΔΔCt^ method. The PCR primers were synthesized by Tsingke Biological Technology (Beijing, China).

### Western blot analysis

Total proteins were extracted from cells and tumor tissues using RIPA buffer supplemented with 1% protease inhibitors on ice and quantified by BCA protein assay kit (Beyotime). Equal amounts of protein samples were separated by SDS-polyacrylamide gel electrophoresis, transferred onto polyvinylidene fluoride membranes (Millipore, Billerica, MA, USA), blocked with 5% non-fat dried milk, and then incubated with the primary antibody overnight at 4 °C. The membranes were incubated with horseradish peroxidase (HRP)-conjugated secondary antibodies for 1 h and detected using an enhanced chemiluminescence kit (Millipore, Billerica, MA, USA). GAPDH was used as the loading control.

The primary antibodies to CDK4 (cat. no. 11026-1-AP), Cyclin D1 (cat. no. 26939-1-AP), Cleaved Caspase 9 (cat. no. 10380-1-AP), Cleaved Caspase 3 (cat. no. 19677-1-AP), Bcl-2 (cat. no. 12789-1-AP), Bax (cat. no. 50599-2-lg), E-cadherin (cat. no. 20874-1-AP), Vimentin (cat. no. 10366-1-AP), MMP2 (cat. no. 10373-2-AP), Nrf2 (cat. no. 16396-1-AP), LC3 (cat. no. 14600-1-AP), p62 (cat. no. 18420-1-AP), Beclin 1 (cat. no. 11306-1-AP), ATG5 (cat. no. 10181-2-AP), and c-Myc (cat. no. 10828-1-AP) were purchased from Proteintech (Rosemont, IL, USA). VEGFR2 (cat. no. AF6281), phosphorylated-VEGFR2 (p-VEGFR2, Tyr1175, cat. no. AF4426), phosphorylated-STAT3 (p-STAT3, Tyr705, cat. no. AF3293), and STAT3 (cat. no. AF6293) were purchased from Affinity (Cincinnati, USA). PD-L1 (cat. no. 13684) was purchased from Cell Signaling Technology (Boston, MA, USA). GAPDH (cat. no. AP0063) was purchased from Bioworld (Nanjing, China).

### Immunofluorescence staining assay

A549 and H1299 cells (1 × 10^5^ cells/well) were plated onto 12-well plates and exposed to apatinib for 48 h. The cells were fixed, permeabilized, blocked with goat serum, and then incubated with antibody against Cleaved Caspase 3 (1:200 dilution, Proteintech), E-cadherin (1:400 dilution, Proteintech), Vimentin (1:400 dilution, Proteintech), p-STAT3 (1:200 dilution, Affinity), Nrf2 (1:200 dilution, Proteintech), p62 (1:200 dilution, Proteintech), and LC3 (1:200 dilution, Proteintech) overnight at 4 °C. Followed by incubation with FITC-conjugated secondary antibody for 45 min, the nuclei were stained with DAPI for 10 min. Finally, the fluorescence images were obtained by a confocal microscopy.

### Tumor xenografts

A 100 μL suspension of 5 × 10^6^ A549 cells were injected subcutaneously into the right axilla of nude mice (SPF grade, 4–5 weeks old, male). Fourteen days after injection, the mice were randomly divided into the control group and apatinib treatment group (*n* = 5 for each group). Apatinib was initially dissolved in DMSO as the stock solution, and was then diluted with saline and the final concentration of DMSO in the oral gavage solution was 2%. The mice in the treatment group were administrated daily with apatinib at 100 mg/kg body weight by oral gavage, while the mice in the control group were administrated with saline solution with equal concentration of DMSO (2%). The body weight of each mouse was measured every 3 day after injection. At the termination of the experiment, the mice were sacrificed and the tumors were removed. The weight, width and length of each tumor was recorded and the tumor volume was calculated using a formula: [width ^2^ × length]/2. Subsequently, tumor tissues were frozen for western blot analysis or embedded for histochemical and immunohistochemistry evaluation.

BALB/c nude mice were purchased from the Animal Research Center of Nanjing Medical University. Animals’ care was performed according to the institutional guidelines, and the animal protocol was approved by the Animal Care and Welfare Committee of Nanjing Medical University (IACUC-1907001).

### Measurement of VEGFR2

Total proteins from mice tumor tissues were lysed using RIPA buffer. The VEGFR2 expression level in tumor tissues was quantified using a mouse Enzyme-Linked Immunosorbent Assay (ELISA) kit followed by the manufacturer’s instructions (Abcam, Cambridge, MA, USA). Optical density was measured at 450 nm, and the amount of VEGFR2 was calculated from a standard curve prepared using the recombinant protein.

### Histochemical or immunohistochemical staining

The tumor tissues were fixed with 4% paraformaldehyde, dehydrated in a series of graded ethanol solutions, and embedded in paraffin for slicing into 5 μm slides. Afterwards, the slides were used for subsequent hematoxylin-eosin (H&E) or immunohistochemical staining. The antibodies used for immunohistochemistry were mouse anti-Cyclin D1 (1:100 dilution, Proteintech), rabbit anti-Cleaved Caspase 3 (1:100 dilution, Proteintech), rabbit anti-E-cadherin (1:100 dilution, Proteintech), rabbit anti-Vimentin (1:100 dilution, Proteintech), rabbit anti-Nrf2 (1:100 dilution, Proteintech), rabbit anti-p62 (1:100 dilution, Proteintech), rabbit anti-LC3 (1:100 dilution, Proteintech), rabbit anti-VEGFR2 (1:100 dilution, Affinity), rabbit anti-p-STAT3 (1:100 dilution, Proteintech), rabbit anti-c-Myc (1:100 dilution, Proteintech), and rabbit anti-PD-L1 (1:100 dilution, Cell Signaling Technology). Eventually, the images were obtained under an optical microscope (Nikon).

### Statistical analysis

Statistical analysis was carried out using GraphPad Prism (Inc., San Diego, CA, USA). The data were presented as mean ± SD. Statistical analysis between two groups were compared using unpaired two-tailed Student’s *t* test. Statistics analysis among multiple groups were compared using one-way analysis of variance (ANOVA). A *P* value < 0.05 was considered as significant difference.

## Results

### Apatinib suppressed cell proliferation in NSCLC cells

**Fig. 1:**
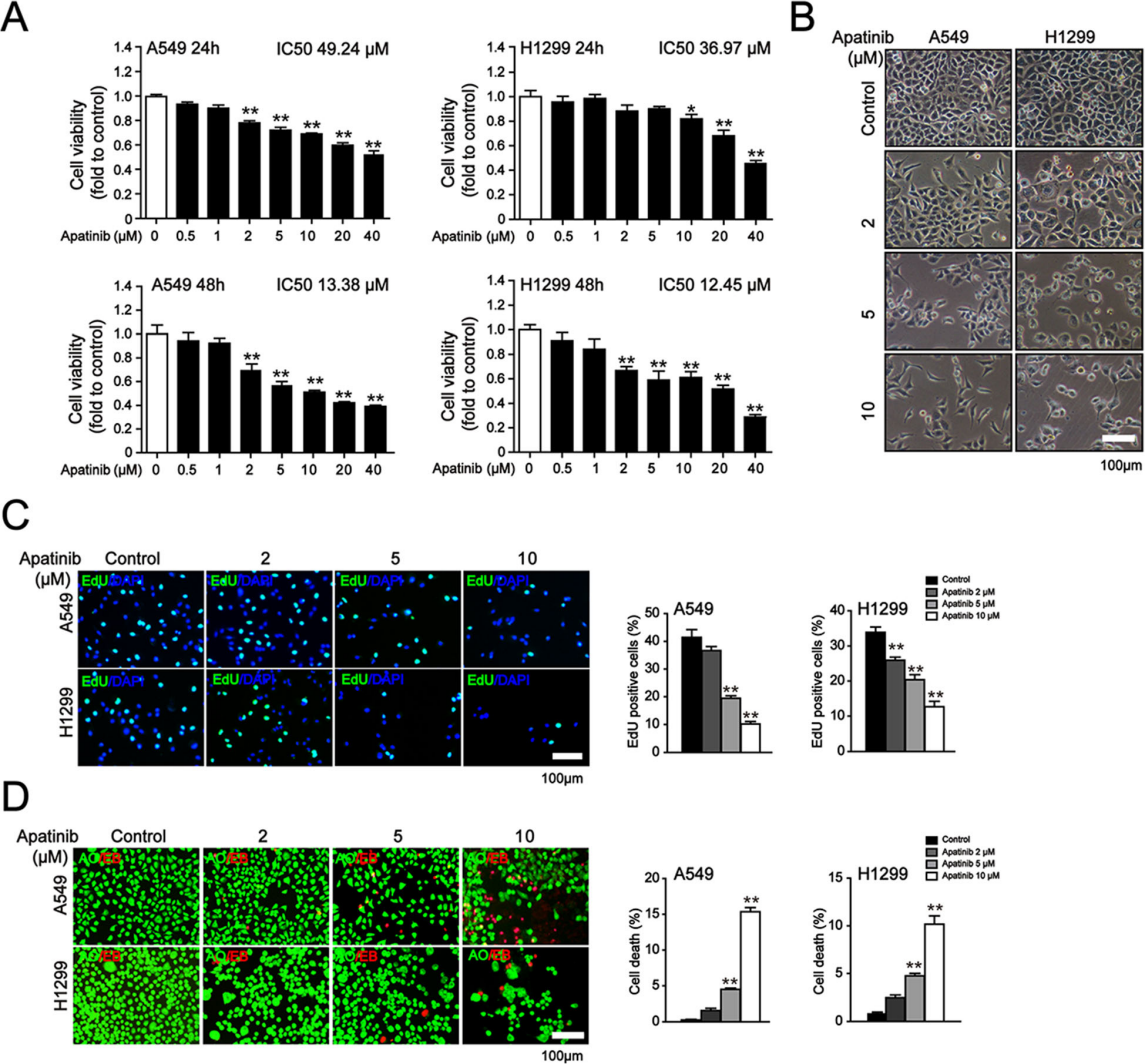
The inhibitory effects of apatinib on the proliferation of NSCLC cells. **a** MTT assay of cell viability in A549 and H1299 cells after indicated apatinib treatment. The data are presented as mean ± SD (*n* = 6). ^*^p < 0.05, ^**^p < 0.01 vs. control. **b** Cell morphology of A549 and H1299 cells after apatinib treatment for 48 h. Scar bar = 100 μm. **c** EdU staining (EdU, green; DAPI, blue) of A549 and H1299 cells after apatinib treatment for 48 h and quantitation of the percentage of EdU-positive cells. Scar bar = 100 μm. The data are presented as mean ± SD (*n* = 3). ^**^p < 0.01 vs. control. **d** AO/EB staining of A549 and H1299 cells after apatinib treatment for 24 h and quantitation of the percentage of cell death. The live cells with normal nuclei appeared green and the dead cells with condensed or fragmented chromatin in the nuclei appeared red. Scar bar = 100 μm. The data are presented as mean ± SD (*n* = 3). ^**^p < 0.01 vs. control

To explore whether apatinib impacted the cell viability and proliferation of NSCLC, A549 and H1299 cells were treated with various concentrations of apatinib for 24 h or 48 h. MTT assay showed that apatinib inhibited cell viability of lung cancer cells in a concertation- and time-dependent manner (Fig. 1a). We observed that apatinib at 2 μM already induced significant inhibition of cell viability in both cells for 48 h treatment, thus, we selected apatinib at concentrations of 2, 5, 10 μM for our further experiments. After 48 h treatment of apatinib, we noticed that A549 and H1299 cells showed round and shrunk morphology and the morphological changes became more obvious with the increasing concentration of apatinib (Fig. 1b). Consistently, apatinib induced a markedly decrease in the percentage of EdU-positive proliferative cells (Fig. 1c). Furthermore, AO/EB staining showed that the death cell numbers were significantly increased in apatinib-treated A549 and H1299 cells (Fig. 1d). Together, these results suggested that apatinib inhibited the growth of NSCLC cells.

### Apatinib arrested cells at G1 phase and induced apoptosis in NSCLC cells

**Fig. 2:**
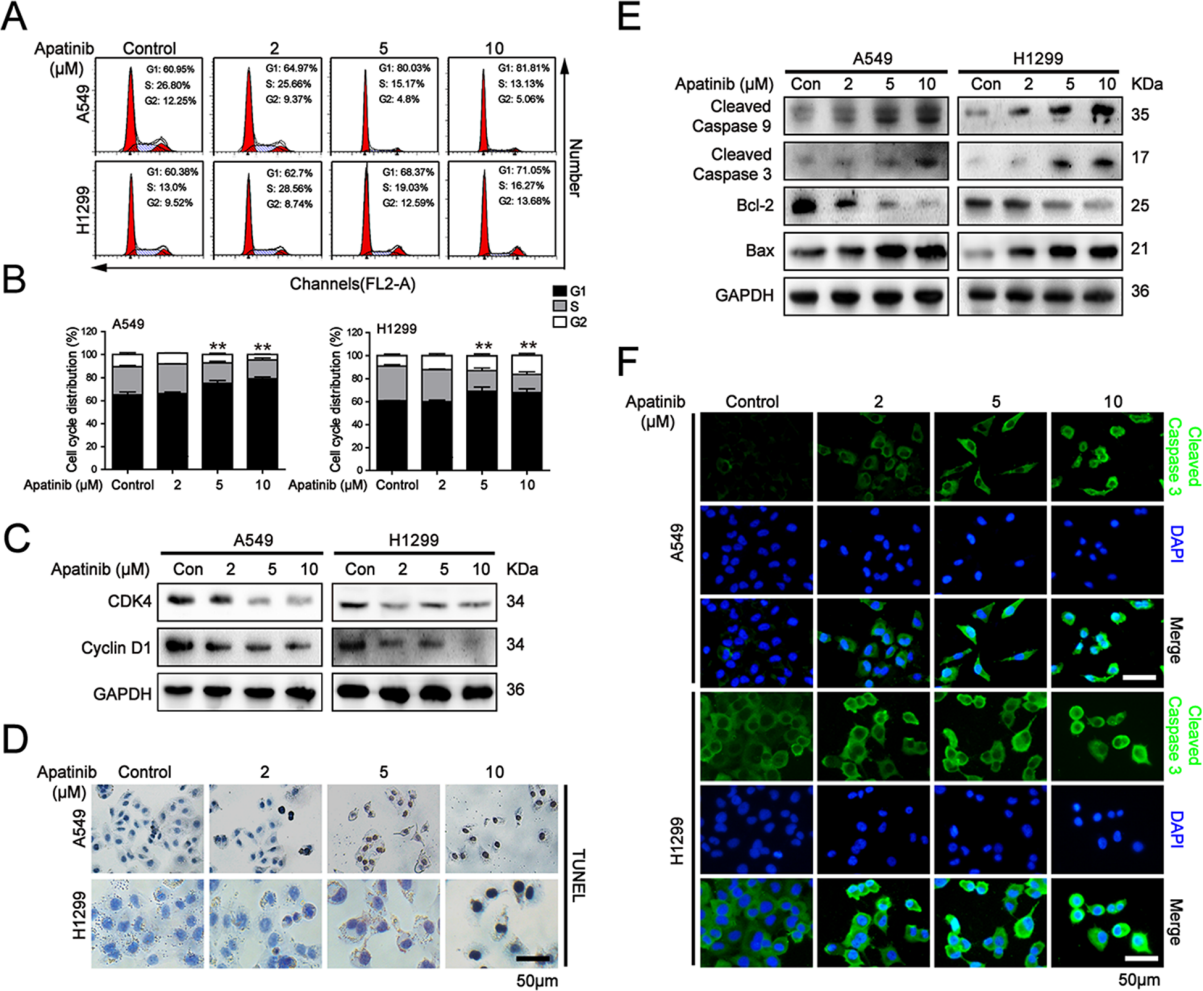
Apatinib induced G1 cell cycle arrest and apoptosis in NSCLC cells. A549 and H1299 cells were treated with indicated treatment for 48 h. **a** Flow cytometry assay of cell cycle distribution. **b** The quantitation of cell cycle distribution. The data are presented as mean ± SD (*n* = 3). ^**^p < 0.01 vs. control. **c** Western blot analysis of G1 phase-related regulators CDK4 and Cyclin D1. **d** TUNEL staining of apoptosis in A549 and H1299 cells. Scar bar = 50 μm. **e** Western blot analysis of apoptosis-related proteins Cleaved Caspase 9, Cleaved Caspase 3, Bcl-2, and Bax expression. **f** Immunofluorescence staining of Cleaved Caspase 3 (Cleaved Caspase 3, green; DAPI, blue). Scar bar = 50 μm

To further investigate the mechanism of apatinib-induced suppression of proliferation in NSCLC cells, cell cycle distributions of A549 and H1299 cells were determined by flow cytometry after apatinib treatment. Our results showed that apatinib at 5 μM and 10 μM blocked the G1 to S cell cycle transition in both cells (Fig. 2a and b). We further analyzed the impact of apatinib on cell cycle related factors. As shown in Fig. 2c, apatinib concentration-dependently downregulated the expressions of G1 phase-regulated proteins CDK4 and Cyclin D1 in A549 and H1299 cells.

Since apoptosis has been thought to be a major antiproliferative mechanism of anticancer drug, we next examined the role of apatinib on apoptosis in NSCLC cells. TUNEL staining assay showed that the rate of apoptotic cells was significantly increased in A549 and H1299 cells upon apatinib treatment (Fig. 2d). Western blot analysis showed that apatinib upregulated the levels of apoptosis proteins Cleaved Caspase 9, Cleaved Caspase 3, and Bax, but downregulated the level of anti-apoptosis protein Bcl-2 (Fig. 2e). Furthermore, immunofluorescence staining analysis also showed the protein expression of Cleaved Caspase 3 was significantly increased in apatinib treated-lung cancer cells (Fig. 2f). Taken together, these results suggested that apatinib induced G1 phase cell cycle arrest and apoptosis in NSCLC cells.

### Apatinib inhibited the malignant and stemness phenotype of NSCLC cells

**Fig. 3:**
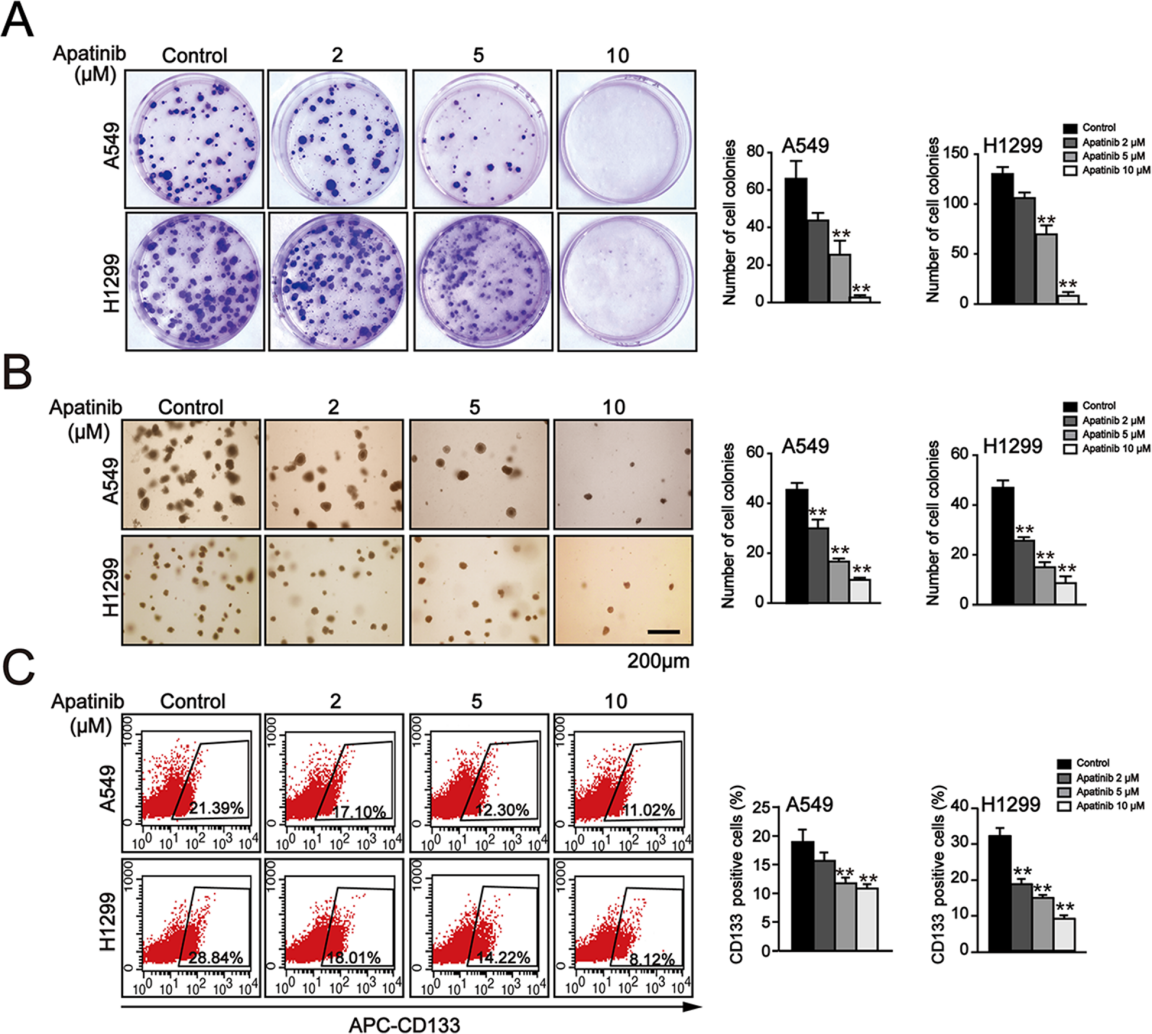
Apatinib inhibited malignant growth and cancer stem cell-like properties in NSCLC cells. **a** Plate colony formation of A549 and H1299 cells after apatinib treatment for 14 days and quantitation of colony numbers. The data are presented as mean ± SD (*n* = 3). ^**^p < 0.01 vs. control. **b** Soft agar colony formation of A549 and H1299 cells after apatinib treatment for 14 days and quantitation of colony numbers. Scar bar = 200 μm. The data are presented as mean ± SD (*n* = 3). ^**^p < 0.01 vs. control. **c** Flow cytometry analysis of CD133-positive cells and quantification of the percentage of CD133-positive cells. The data are presented as mean ± SD (*n* = 3). ^**^p < 0.01 vs. control

To analyze the role of apatinb on the malignant phenotype of NSCLC, plate and soft agar colony formation assays were performed. We found that the plate colony formation ability of both A549 and H1299 cells was decreased in a concentration-dependent manner after apatinib treatment (Fig. 3a). Meanwhile, soft agar colony formation assay also showed a similar inhibition of apatinib on anchorage-independent growth capability of NSCLC cells (Fig. 3b).

The cancer stem cell hypothesis posits that the existence of cancer stem cells is a vital mechanism for cancer initiation, development, and chemoresistance. CD133 is recognized as a specific surface marker of lung cancer stem cells and CD133-positive cells exhibit stem cell-like properties [28, 29]. We further investigated the effect of apatinib on the stemness phenotype of NSCLC cells. A549 and H1299 cells were cultured with indicated concentrations of apatinib in a stem cell specific serum-free medium for 7 days and the numbers of CD133-positive cells were detected by flow cytometry assay. As shown in Fig. 3b, the percentage of CD133-positive cells was significantly downregulated in apatinib treated-cells. These results suggested that apatinib inhibited malignant growth and cancer stem cell-like properties in NSCLC cells.

### Apatinib suppressed the migration and invasion capacities of in NSCLC cells

**Fig. 4:**
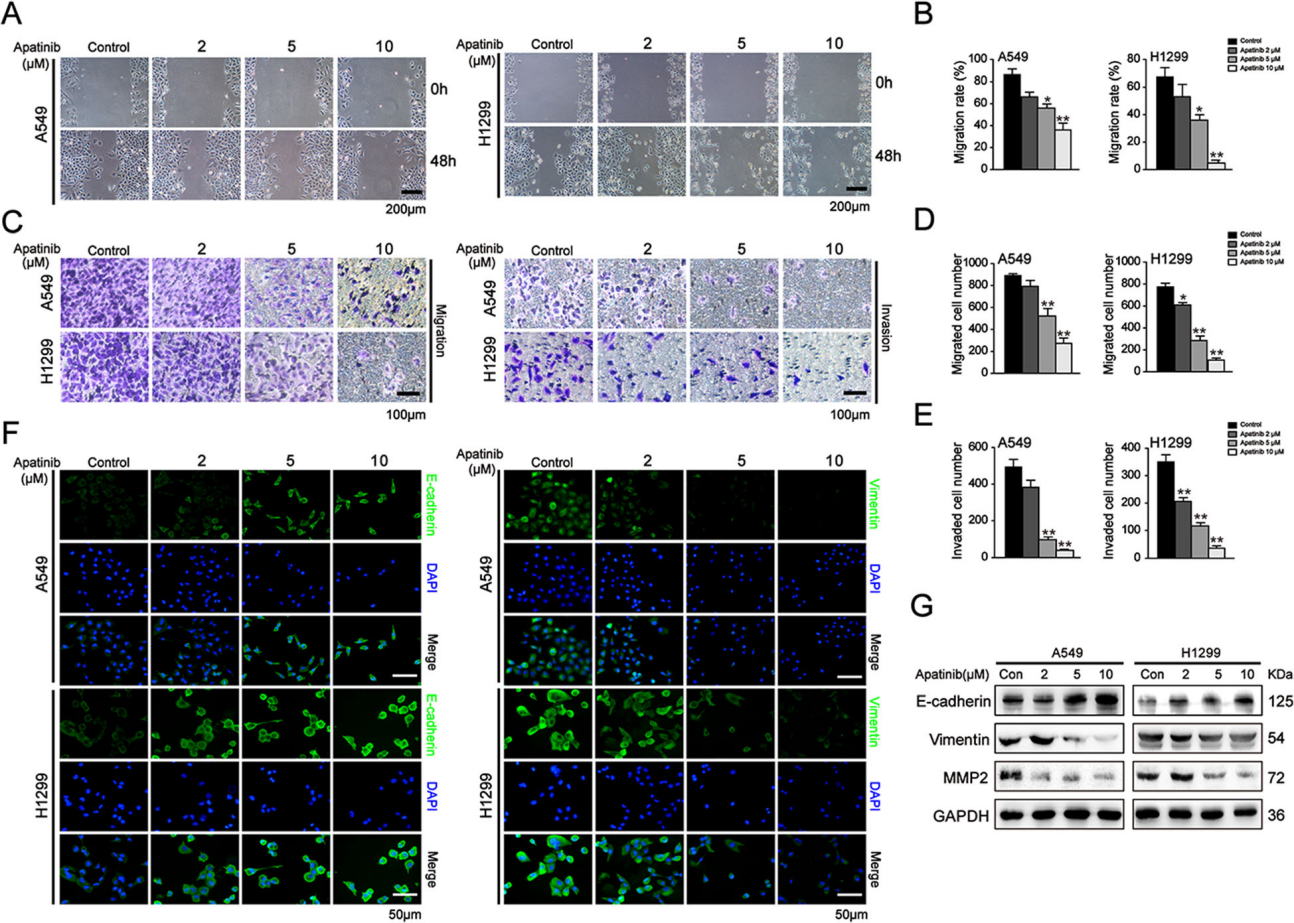
Apatinib suppressed the migration and invasion of NSCLC cells. A549 and H1299 cells were treated with apatinib for 48 h. **a** and **b** Wound healing assay was performed to evaluate the migration capacity. The migration rate was quantitated. Scar bar = 200 μm. The data are presented as mean ± SD (*n* = 3). ^*^p < 0.05, ^**^p < 0.01 vs. control. **c-e** Transwell assays without or with Matrigel were performed to evaluate the migration and invasion capacity. The numbers of migrated and invaded cells were quantitated. Scar bar = 100 μm. The data are presented as mean ± SD (*n* = 3). ^*^p < 0.05, ^**^p < 0.01 vs. control. **f** Immunofluorescence staining of E-cadherin and Vimentin (E-cadherin and Vimentin: green; DAPI: blue). Scar bar = 50 μm. **g** Western blot analysis of EMT-regulated proteins E-cadherin, Vimentin and MMP2

To assess the influence of apatinib on the metastatic potency of NSCLC cells, wound healing and transwell assays were carried out. As shown in Fig. 4a and b, apatinib retarded wound closure in a concertation-dependent manner. Transwell assay further showed that after 48 h treatment of apatinib, the numbers of migrated cells were significantly reduced when compared to that of the control cells. In addition, the numbers of A549 and H1299 cells that invaded onto the lower chamber through extracellular matrix gels were also markedly decreased following apatinib treatment (Fig. 4c-e). These results indicated that apatinib effectively suppressed the migration and invasion capacity of NSCLC cells.

Given the epithelial-mesenchymal transition (EMT) is the initiating step towards invasion and distant metastasis, we next investigated the expression of EMT-related markers upon apatinib treatment. Immunofluorescence staining analysis showed that apatinib upregulated the expression of epithelial-related marker E-cadherin and concomitantly downregulated the expression of mesenchymal-related marker Vimentin in A549 and H1299 cells (Fig. 4f). Moreover, Western blotting further confirmed that the protein level of E-cadherin was increased, whereas the levels of Vimentin and MMP2 were decreased in apatinib treated-cells (Fig. 4g). Taken together, these results indicated that apatinib inhibited metastasis by repressing the EMT process in NSCLC cells.

### Apatinib downregulated VEGFR2/STAT3/PD-L1 signaling in NSCLC cells and reduced the immunosuppressive TME

**Fig. 5:**
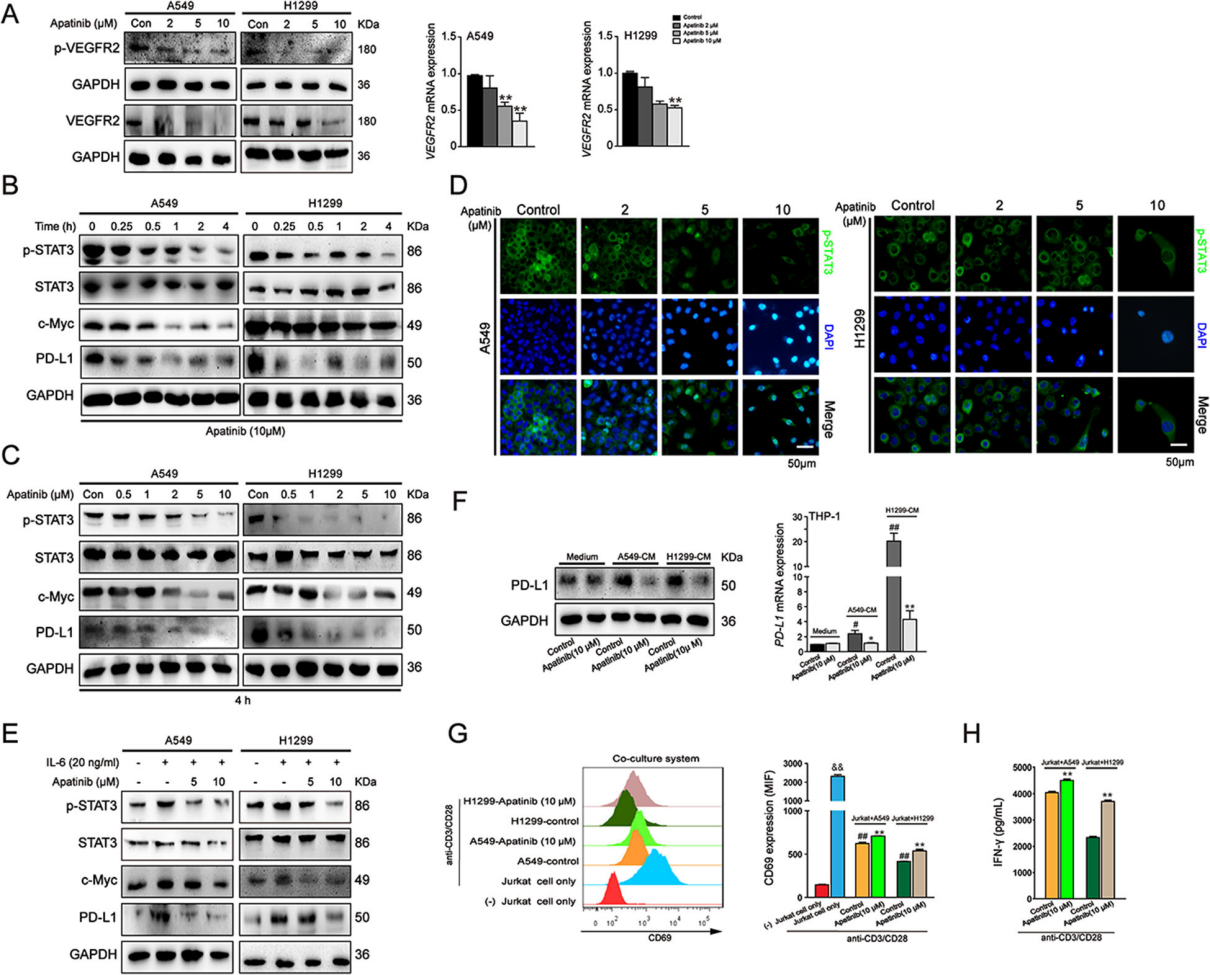
Apatinib downregulated VEGFR2/STAT3/PD-L1 pathway in NSCLC cells and reduced the immunosuppressive TME. **a** Western blot analysis of VEGFR2 and p-VEGFR2 (Tyr1175) expression and qRT-PCR analysis of VEGFR2 expression in A549 and H1299 cells after aptinib treatment for 48 h. The data are presented as mean ± SD (*n* = 3). ^**^p < 0.01 vs. control. **b** and **c** Western blot analysis of p-STAT3, STAT3, c-Myc and PD-L1 expressions in A549 and H1299 cells after indicated treatment. **d** Immunofluorescence staining of p-STAT3 in A549 and H1299 cells after apatinib treatment for 4 h (p-STAT3: green; DAPI: blue). Scar bar = 50 μm. **e** Western blot analysis of p-STAT3, STAT3 and PD-L1 expressions in A549 and H1299 cells after apatinib treatment with or without pretreatment of IL-6 for 48 h. **f** Western blot and qRT-PCR analysis of PD-L1 expression in THP-1-derived macrophages with or without stimulation with CM from A549 and H1299 cells. The data are presented as mean ± SD (*n* = 3). ^*^p < 0.05, ^**^p < 0.01 vs. A549 or H1299-CM control. ^#^*p* < 0.05, ^#^*p* < 0.01 vs. medium control. **g** and **h** Jurkat cells were activated by stimulation with anti CD3/CD28 antibodies and then co-cultured with non-treated or apatinib (10 μM) pretreated A549 or H1299 cells; the Jurkat cells were collected for CD69 detection by flow cytometry (**g**) and the co-culture medium was collected for IFN-γ secretion by ELISA assay (**h**). The data are presented as mean ± SD (*n* = 3). ^&&^ *p* < 0.01 vs (−) Jurkat cell only group. ^**^p < 0.01 vs. A549 + Jurkat or H1299 + Jurkat control group. ^##^ *p* < 0.01 vs. Jurkat cells only stimulated with anti CD3/CD28 antibodies group

It is acknowledged that apatinib highly selectively targets VEGFR2 and blocks VEGF-induced proliferation and migration of endothelial cells. Previous studies have demonstrated that apatinib exerts antitumor abilities in multiple solid tumors by targeting VEGFR2/STAT3 pathway. Accordingly, we explored whether apatinib inhibited NSCLC cells by regulating VEGFR2/STAT3 signaling pathway. As shown in Fig. 5a, after 48 h treatment, apatinib significantly downregulated the protein and mRNA expression of VEGFR2 and the levels of p-VEGFR2 (Tyr1175) in A549 and H1299 cells. Moreover, our results showed that the protein level of p-STAT3 was time- and concentration-dependently decreased in apatinib-treated cells in comparison with that in the control cells (Fig. 5b and c). Immunofluorescence staining assay also showed that apatinib markedly suppressed p-STAT3 expression (Fig. 5d). Additionally, the protein levels of c-Myc and PD-L1, the major downstream targets of STAT3 signaling displayed a consistent decline upon apatinib treatment (Fig. 5b and c). To further analyze whether apatinib inhibited NSCLC cells via STAT3 signaling, we treated A549 and H1299 cells with apatinb after pretreatment of 20 ng/mL interleukin-6 (IL-6), a major mediator of inflammation and activator of STAT3. The results showed that IL-6 induced the activation of STAT3 pathway, while the expression levels of IL-6 upregulated-p-STAT3, c-Myc, and PD-L1 were reversed by apatinib (Fig. 5e).

To further explore whether apatinib could impact PD-L1 expression in tumor-associated macrophages, we treated THP-1 derived macrophages with 10 μM apatinib for 4 h after stimulation of CM from A549 and H1299 cells. Western blot and qRT-PCR analysis showed that apatinib markedly inhibited the protein and mRNA level of PD-L1 in CM-treated macrophages, but had no obvious impact on PD-L1 expression in macrophages without CM stimulation (Fig. 5f). The results indicated that apatinib inhibited tumor-associated macrophages in TME.

We further examined whether apatinib downregulated PD-L1 in NSCLC and THP-1 cells was associated with reduced immunosuppressive activity of these cells against T-cells. Thus, we established Jurkat T cells and A549 or H1299 cells co-culture model. As CD69 is the earliest cell surface marker of activated T cells [30], we then examined the effect of apatinib on the expression of CD69 in activated Jurkat T cells. As shown in Fig. 5g, Jurkat T cells exhibited elevated CD69 level after stimulation with anti CD3/CD28 antibodies, and co-culture of activated Jurkat T cells with A549 or H1299 cells significantly decreased its expression. As expected, the level of CD69 was upregulated in activated Jurkat T cells when co-cultured with apatinib (10 μM) pretreated NSCLC cells when compared with non-treated control cells (Fig. 5g). We also examined the secretion of IFN-γ, which was produced by the effector T cells in the co-culture cell medium. Our results showed that pretreatment of apatinib increased IFN-γ production in activated Jurkat T cells in comparison with non-treated control cells (Fig. 5h). Taken together, our results suggested that apatinib partially restored the activation of Jurkat T cells and reduced the immunosuppressive TME.

### Apatinib induced autophagic and apoptotic cell death via ROS/Nrf2/p62 pathway in NSCLC cells

**Fig. 6:**
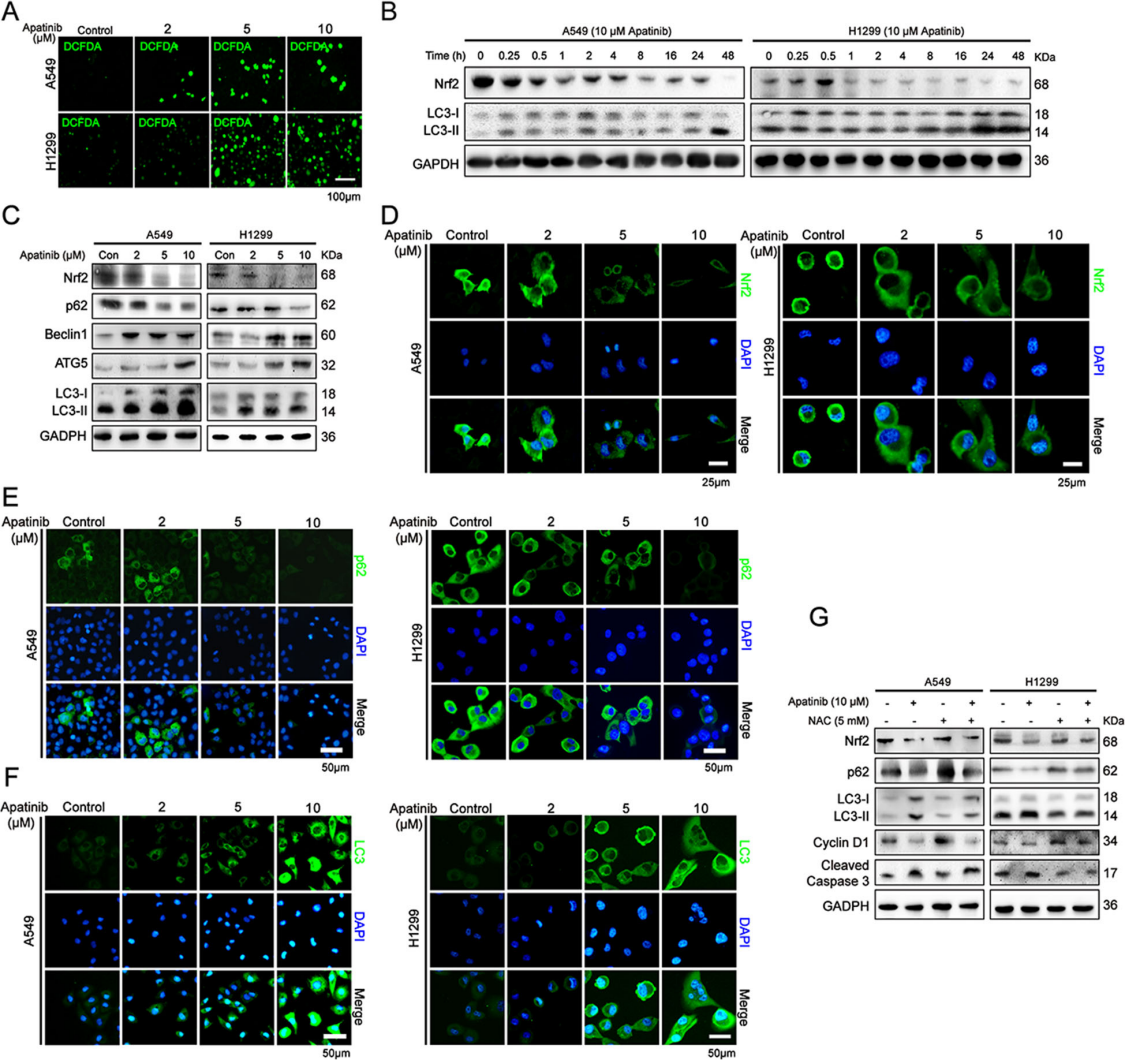
Apatinib induced autophagic and apoptotic cell death via ROS/Nrf2/p62 pathway in NSCLC cells. **a** The representative images of fluorescence intensity of DCFH-DA in apatinib-treated A549 and H1299 cells. Scale bar = 100 μm. **b** Western blot analysis of Nrf2 and LC3 expressions in apatinib (10 μM)-treated A549 and H1299 cells for 0–48 h. **c** Western blot analysis of Nrf2, p62, Beclin1, ATG5, and LC3 expressions in A549 and H1299 cells after various concentrations of apatinib treatment for 48 h. **d-f** Immunofluorescence staining of Nrf2 (**d**), p62 (**e**), and LC3 (**f**) in A549 and H1299 cells after indicated treatment (Nrf2, p62, and LC3: green; DAPI: blue). Scar bar = 50 μm. **g** Western blot analysis of Nrf2, p62, LC3, Cyclin D1 and Cleaved Caspase 3 expressions in A549 and H1299 cells after apatinib with or without NAC treatment for 48 h

Previous studies demonstrated that anticancer drugs triggered apoptosis of tumor cells by inducing the excessive generation of ROS. We examined the role of apatinib on ROS production in NSCLC cells. As shown in Fig. 6a, after 24 h treatment of apatinib, the production of ROS in A549 and H1299 cells were significantly increased. Since the antioxidant system determines the cellular level of ROS, we further evaluated the expression of the antioxidant enzyme Nrf2 after apatinib treatment. We observed that short time (0.25 h and 0.5 h) treatment of 10 μM apatinib did not significantly inhibit Nrf2 expression in A549 cells, whereas Nrf2 expression was increased in apatinib-treated H1299 cells for 0.5 h. With the prolong of apatinib treatment, the protein level of Nrf2 was eventually decreased in both cells (Fig. 6b). Western blot and immunofluorescence staining assays further showed that Nrf2 expression was decreased in a concentration-dependent manner in apatinib treated-cells (Fig. 6c and d). In addition, the inhibition of apatinib on Nrf2 expression was obviously reversed by NAC, a ROS scavenger (Fig. 6g). These results suggested that apatinib inhibited NSCLC by inducing ROS production and disrupting antioxidant defense system.

We next explored whether apatinib-induced ROS production caused autophagy in NSCLC. It is reported that in the process of autophagy, the cytosolic LC3-I is converted to autophagic vesicle-associated LC3-II. Our results showed that the expression level of LC3-II form was markedly upregulated after apatinib treatment (Fig. 6b, c and f). Additionally, an increase in the expressions of autophagy related-proteins Beclin1 and ATG5 and a decrease in p62 expression were significantly detected in apatinib treated-A549 and H1299 cells (Fig. 6c and e). Furthermore, our results showed that NAC treatment obviously attenuated apatinib-induced autophagy and apoptosis. As shown in Fig. 6g, apatinib downregulated-Nrf2, p62, and Cyclin D1 and upregulated-LC3-II and Cleaved Caspase 3 were partially reversed by NAC. Collectively, these results suggested that apatinib induced autophagic and apoptotic cell death through ROS/Nrf2/p62 pathway.

### Apatinib inhibited the growth of NSCLC cells in vivo

**Fig. 7:**
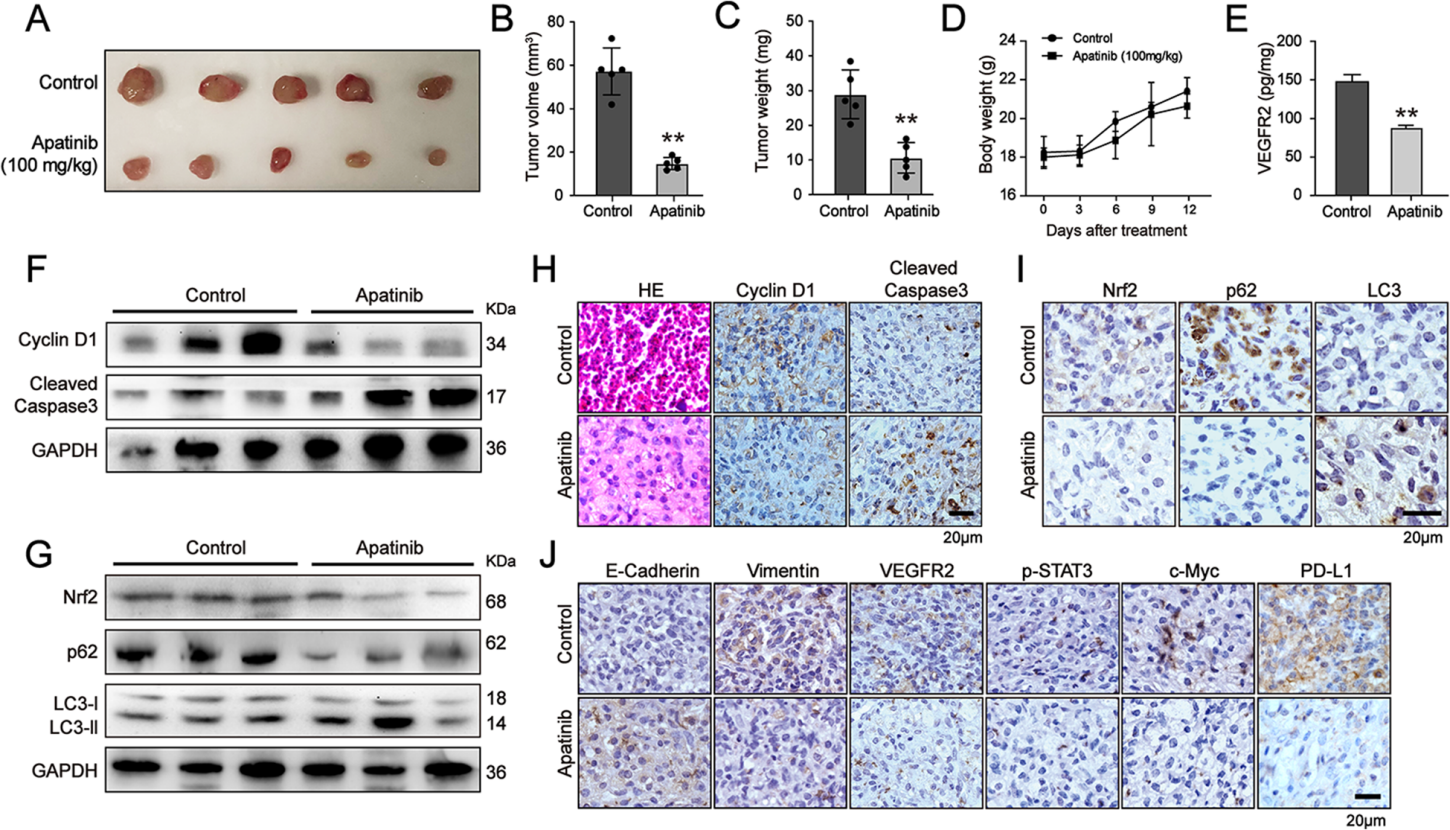
Apatinib inhibited the growth of NSCLC cells in vivo. **a** Images of the xenograft tumors of the control and apatinib treatment group. **b** and **c** Tumor volume and tumor weight were measured. **p < 0.01 vs. control. The data are presented as mean ± SD (*n* = 5). ^**^p < 0.01 vs. control. **d** Body weights of mice were recorded every 3 days after indicated treatment. The data are presented as mean ± SD (*n* = 5). **e** ELISA assay of VEGFR2 expression in tumors. The data are presented as mean ± SD (*n* = 5). ^**^p < 0.01 vs. control. **f** and **g** Western blot analysis of Cyclin D1, Cleaved Caspase 3, Nrf2, p62, and LC3 expressions in tumors. **h-j** H&E staining of tumors and immunohistochemical staining of Cyclin D1, Cleaved Caspase 3, Nrf2, p62, LC3, E-cadherin, Vimentin, VEGFR2, p-STAT3, c-Myc, and PD-L1 expression in the tumors

To investigate the effects of apatinib on tumor growth in vivo, A549 cells were inoculated into BALB/c nude mice by subcutaneous injection Fourteen days after injection, the mice were randomly divided into the control group and apatinib treatment group. The mice in the treatment group were orally administrated daily with apatinib at 100 mg/kg body weight, while the mice in the control group were administrated with vehicle solution. After administration for another 12 days, both tumor volume and tumor weight were significantly reduced in apatinib-treated mice (Fig. 7a-c), indicating the inhibitory effects of apatinib on tumor growth in vivo. Meanwhile, we also measured the body weight of each mouse during the experiment. We found that there were no obvious changes in the body weight of mice after apatinib administration, suggesting that the tolerance and efficiency of apatinib treatment (Fig. 7d).

Moreover, we showed that apatinib inhibited proliferation and induced apoptotic and autophagic death in lung cancer tumors, as evidenced by the decreased expression of Cyclin D1, Nrf2 and p62, but the increased expression of Cleaved Caspase 3 and LC3-II (Fig. 7f-i). Immunohistochemical staining analysis also showed that apatinib upregulated the level of E-cadherin and downregulated the level of Vimentin (Fig. 7j). ELISA and immunohistochemical staining analysis confirmed that apatinib markedly reduced VEGFR2 level in tumors (Fig. 7e and j). Immunohistochemical staining results further showed that the protein levels of p-STAT3, c-Myc, and PD-L1 were also downregulated in apatinib-treated tumors (Fig. 7j). Taken together, consistent with in vitro studies, our results suggested that apatinib inhibited the tumor growth of NSCLC cells by targeting both Nrf2 and VEGFR2/STAT3 signaling in vivo.

## Discussion

**Fig. 8:**
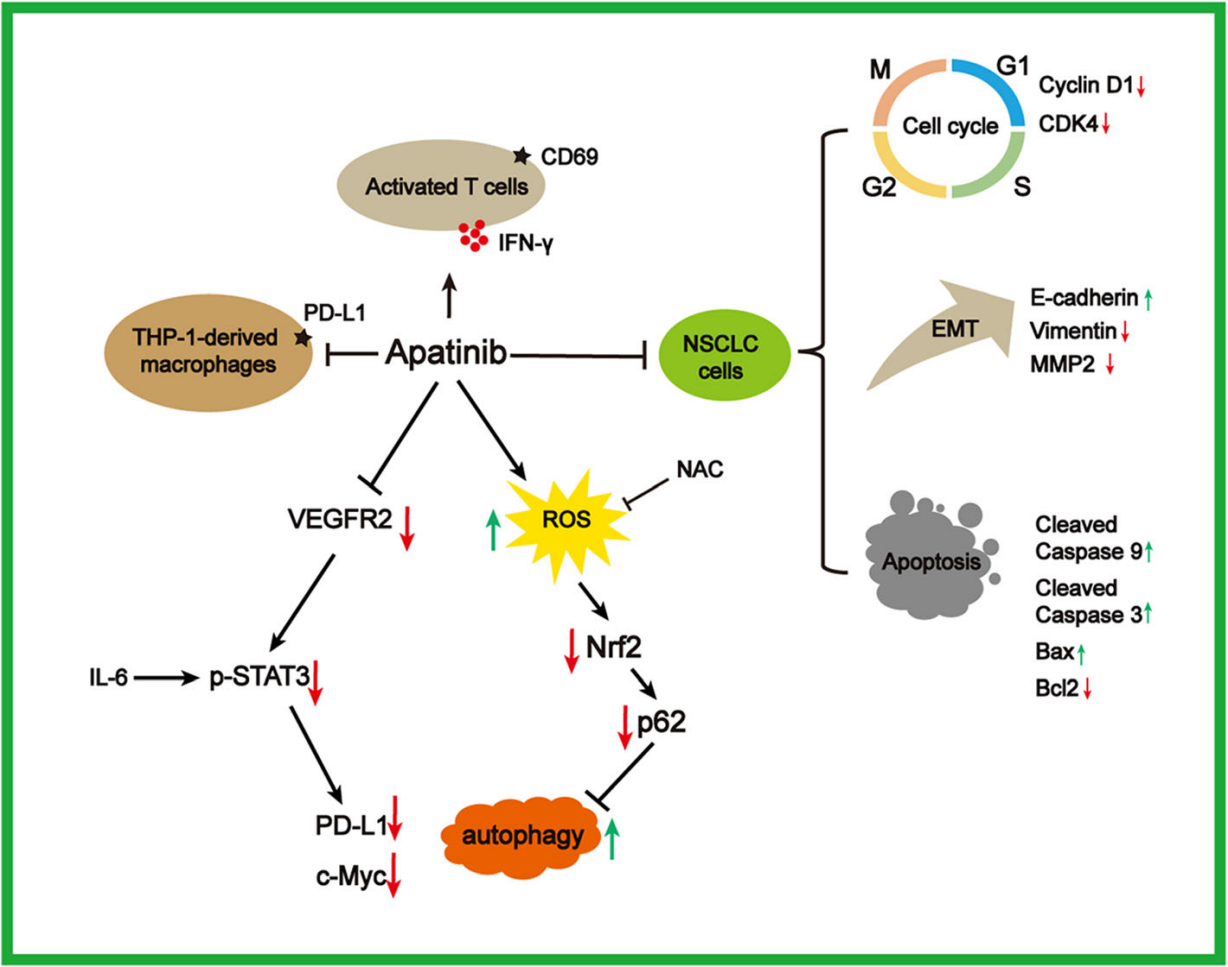
Schematic diagram of how apatinib inhibited NSCLC. Apatinib suppressed cell proliferation, induced cell cycle arrest and apoptosis, and inhibited malignance of NSCLC cells. Mechanistically, apatinib downregulated PD-L1 and c-Myc expression through targeting VEGFR2/STAT3 pathway and induced ROS-triggered autophagy via decreasing Nrf2 and p62 in NSCLC cells. Apatinib further reduced immunosuppressive TME by suppressing PD-L1 expression in tumor-associated macrophages and partially restoring the activation of T cells

Although enormous efforts have been made, the prognosis and overall survival of patients with NSCLC have not been improved yet. Considering NSCLC is a a highly vascularized tumor, angiogenesis blockade has been identified as an effective therapeutic strategy in inhibition of lung cancer progression. Apatinib is a highly selective TKI to VEGFR2 and has become an attractive drug in the treatment of NSCLC. However, the underlying mechanism of apatinib on NSCLC requires further illustration, which is critical for the clinical translational application of apatinib. A large number of studies have indicated that apatinib directly suppressed cell growth and triggered apoptosis in wide range of cancers [17, 25, 26, 31–33]. Consistent with the previous findings, we first observed the antitumor effects of apatinib on NSCLC A549 and H1299 cells in vitro and in vivo. Our results further showed that apatinib reduced the expression levels of phosphorylated STAT3 and its major downstream targets (c-Myc and PD-L1) in a concentration- and time-dependent manner. Meanwhile, apatinib treatment induced autophagic and apoptotic cell death by promoting ROS generation and inhibiting Nrf2 and p62 expression. Based on the multi-faceted evidence, we proposed a regulatory pathway of apatinib on proliferation inhibition and apoptosis induction in NSCLC (Fig. 8).

In the present study, we first observed that apatinib significantly inhibited cell viability and proliferation in NSCLC cells in vitro. Our results further showed that apatinib suppressed the growth of A549 and AH299 cells by inducing G1 phase cell cycle arrest and apoptosis, which was also reported in PC9 gefitinib-resistant cancer cells [34]. Liu et al. reported that apatinib resensitized cisplatin-resistant A549 cells by increasing the levels of Cleaved Caspase 3, Cleaved Caspase 9 and Bax and decreasing Bcl-2 expression [35]. Our data also showed that apatinib had a similar impact on apoptosis in A549 and H1299 cells. Therefore, these results indicated that apatinib inhibited the growth of NSCLC cells through cell cycle arrest and apoptosis. Many studies have reported that A549 cells expressed a relative low level of VEGFR2 among NSCLC cell lines [36–38]. Our results presented evidence that NSCLC cells expressing low levels of VEGFR2 still responded to apatinib.

A previous report showed that apatinib suppressed the stemness of lung cancer stem cells in vivo by downregulating Hedgehog, Hippo, and Wnt pathways [39]. Our results also indicated that apatinib directly decreased the percentage of CD133-positive cells in A549 and H1299 cells culturing in a specific stem cell medium in vitro. It is acknowledged that CD133 is an important marker of lung cancer stem cells and NSCLC prognosis [40, 41]. Simultaneously, numerous studies have demonstrated that apatinib inhibited migration and invasion in various tumors, including liver cancer [33], osteosarcoma [17], and gastric cancer [42]. Our study showed that apatinib markedly inhibited the migration and invasion capability of A549 and H1299 cells. Furthermore, we also showed that apatinib negatively regulated EMT-related markers, including E-cadherin, Vimentin and MMP2. Taken together, combined previous studies with our results, apatinib was considered as a promising therapeutic drug for NSCLC.

Recent studies have shown that the antitumor mechanisms of apatinib were associated with various multiple signaling pathways and specific regulatory molecules. In osteosarcoma, apatinib exerted its antitumor effects via VEGFR2/STAT3/BCL-2 pathway [26]. Apatinib inhibited osteosarcoma by targeting STAT3 and reducing PD-L1/PD-L2 [17, 43]. Furthermore, apatinib suppressed doxorubicin-induced stemness phenotype in osteosarcoma cells via STAT3 signaling [44]. In ovarian, liver and leukemia caner, apatinib regulated cell proliferation, apoptosis and EMT by inhibiting VEGFR2/STAT3 pathway [31, 32, 45]. Our results showed that apatinib (5 μM and 10 μM) induced a significant reduction of the total VEGFR2 level and had obvious cytotoxic effects in NSCLC cells. The cytotoxic effects of apatinib might lead to the reduction of total VEGFR2 in NSCLC cells. It is reported that multiple signaling pathways and molecules might be involved in the regulation of VEGFR2 expression [46–48]. Our previous study also indicated that apatinib suppressed lung cancer stem-like properties by inactivation of β-catenin signaling, which might further lead to the downregulation of VEGF and its receptor VEGFR2 [49]. STAT3 plays an essential role in tumor development and has been considered as a promising target for cancer treatment, especially in NSCLC therapy [50]. Meanwhile, STAT3 is constitutively hyper activated in EGFR-mutated NSCLC. In our study, we also found that apatinib decreased phosphorylated STAT3 in NSCLC in vitro and in vivo. Li et al. found that apatinib markedly potentiated the antitumor effect of gefitinib in NSCLC with T790M-related EGFR-TKI resistance [51]. Several studies demonstrated that apatinib plus EGFR-TKIs (gefitinib) exhibited a tolerable safety profile and encouraging antitumor activity for advanced EGFR-mutant NSCLC [52–54]. Thus, the efficacy of apatinib in EGFR-mutant NSCLC might be related with the inactivation of STAT3. Our results suggest that apatinib may be an alternative therapeutic approach for patients with advance lung cancer.

We further observed that PD-L1 expression was also decreased in apatinib-treated NSCLC cells. PD-L1, one of the hot spots in current immunotherapy, can be induced by the intrinsic oncogenic pathways, including STAT3 signaling [55]. Overexpression of PD-L1 was contributed to the poor prognosis of lung cancer [56]. A recent study illustrated that low-dose apatinib with anti-PD-L1 antibody optimized tumor microenvironment by alleviating hypoxia, promoting the infiltration of CD8 (+) T cells, reducing recruitment of tumor-associated macrophages in lung cancer [15]. STAT3 pathway was reported to be the most critical downstream of IL-6 signaling that modulated PD-L1 in lung cancer cells. It has been reported that IL-6 regulated inflammation and the immune response by promoting the phosphorylation of STAT3 [57]. We noted that apatinib not only inhibited IL-6-mediated upregulation of phosphorylated STAT3 and PD-L1 in NSCLC cells, but also suppressed PD-L1 activation in THP-1- derived macrophages which were stimulated by CM from A549 and H1299 cells. We also found that pretreatment of apatinib partially restored the activation of Jurket T cells co-cultured with NSCLC cells, as evidenced by the increase of CD69 expression and IFN-γ secretion. Therefore, our results indicated that apatinib might exert its inhibitory effects on NSCLC by targeting VEGFR2/STAT3/PD-L1 signaling in both NSCLC cells and tumor derived macrophages and partially restoring theactivation of T cells. Collectively, our data suggested that apatinib might reduce the immunosuppressive TME in NSCLC.

Traditional chemotherapeutic drugs used in cancer therapy generally induced ROS production and disturbed the intracellular reduction capacity, resulting in the induction of cell death [58]. Apatinib has been reported to promote ROS-dependent apoptosis in many cancer cells, such as ovarian cancer cells [24] and breast cancer cells [59]. Our results showed that apatinib induced ROS production in NSCLC A549 and H1299 cells. Our previous study found that apatinib-triggered ROS induced mitochondrial transmembrane potential reduction, leading to the mitochondrial dysfunction [49]. VEGFR2-downstream signaling is linked to mitochondria biology in cancer [60, 61], which might be involved in apatinib-triggered ROS. It is reported that the transcriptional factor Nrf2 regulates the expression of antioxidant genes and plays a vital role in redox homeostasis. Emerging evidence has shown that Nrf2 pathway is frequently dysregulated in lung cancer and overactivation of the pathway is associated with poor prognosis of NSCLC [62, 63]. Our results indicated that apatinib inhibited Nrf2 expression in NSCLC in a concentration- and time-dependent manner. Consistent with our results, Sun et al. found that apatinib downregulated Nrf2 and promoted ROS production in ovarian cancer [24]. Therefore, we speculated that apatinib promoted ROS generation to inhibit NSCLC by targeting Nrf2 and ROS exerted a critical role in apatinib-induced apoptosis in NSCLC.

Of note, autophagy is very important in regulating cell survival and homeostasis. It has been demonstrated that autophagy and apoptosis interact with each other to induce cell death. Previous studies showed apatinib promoted autophagy and apoptosis in many tumor types [26]. However, litter is known about the role of apatinib on autophagy in NSCLC. In our present study, we showed that apatinib significantly enhanced autophagic and apoptotic cell death in NSCLC in vitro and in vivo. It has been demonstrated that autophagy and the Nrf2 system are the major cellular defense mechanisms against oxidative stress. These two systems affect each other via p62 (autophagy receptor protein) and Keap1 (the Nrf2 substrate adaptor for the Cul3 E3 ubiquitin ligase) [64]. Our results indicated that pretreatment of antioxidant NAC reversed apatinib-downregulated Nrf2 and -upregulated autophagy. We further showed that the effects of apatinib on cell proliferation and apoptosis were attenuated by NAC. Therefore, these data indicated that apatinib induced ROS production and reduced Nrf2 and p62 expression, which further led to the autophagy and apoptosis of NSCLC cells.

## Conclusion

In conclusion, our results showed that apatinib suppressed proliferation and promoted autophagic and apoptotic cell death of NSCLC by regulating both VEGFR2/STAT3/PD-L1/c-Myc and ROS/Nrf2/p62 signaling. Findings from the present study indicated that apatinib might be a promising therapeutic agent for NSCLC.

## Supplementary Information

**Additional file 1:Supplementary**. Raw data for the blots.

## Data Availability

The datasets supporting the conclusions of this article are included within the article.

## Abbreviations

NSCLC: Non-small cell lung cancer
VEGF: Vascular endothelial growth factor
VEGFR: Vascular endothelial growth factor receptor
TKI: Tyrosine kinase inhibitor
TME: Tumor microenvironment
PD-1: Programmed death 1
PD-L1: Programmed death ligand 1
STAT: Signal transducer and activator of transcription
Nrf2: Nuclear factor erythroid-derived 2-like 2
ROS: Reactive oxygen species
Keap1: Kelch-like ECH-associated protein 1
NAC: N-acetyl-L-cysteine
CM: Conditioned-medium
IFNγ: Interferon-γ
EMT: Epithelial-mesenchymal transition
IL-6: Interleukin-6
