# Correction to: Apatinib triggers autophagic and apoptotic cell death via VEGFR2/STAT3/PD-L1 and ROS/Nrf2/p62 signaling in lung cancer

**DOI:** 10.1186/s13046-021-02142-y

**Published:** 2021-11-06

**Authors:** Chunfeng Xie, Xu Zhou, Chunhua Liang, Xiaoting Li, Miaomiao Ge, Yue Chen, Juan Yin, Jianyun Zhu, Caiyun Zhong

**Affiliations:** 1 Department of Nutrition and Food Safety, School of Public Health, Nanjing Medical University, 101 Longmian Ave, Jiangning, Nanjing, 211166 China; 2 Department of Laboratory, The Affiliated Suzhou Hospital of Nanjing Medical University, Suzhou Municipal Hospital, Gusu School, Nanjing Medical University, 242 Guangji Rd, Suzhou, 215008 China; 3 Cancer Research Division, Center for Global Health, School of Public Health, Nanjing Medical University, Nanjing, 211166 Jiangsu China

**Correction to: J Exp Clin Cancer Res 40, 266 (2021)**

**https://doi.org/10.1186/s13046-021-02069-4**

Following publication of the original article [1], minor errors were identified in the images presented in Figs. 2, 4, and 5; specifically:

- Fig. 2a: Flow cytometry assay of cell cycle distribution; bottom left panel has been corrected (incorrect ‘S’ percentage originally displayed)
- Fig. 4a: Wound healing assay for H1299 cells after apatinib treatment for 48h; correct photograph used for Stage 5 (2^nd^ row, 3^rd^ column)
- Fig. 5d: Immunofluorescence staining of p-STAT3 in A549 cells after apatinib treatment; correct photograph used for Stage 2 Merge (3^rd^ row, 2^nd^ column)
- Fig. 5d: Immunofluorescence staining of p-STAT3 in H1299 cells after apatinib treatment; correct photograph used for Stage 5 p-STAT3 (1^st^ row, 3^rd^ column)

The authors provided the journal with the original data files. The corrected figures are given below. The correction does not have any effect on the results or conclusions of the paper. The original article has been corrected.

**Fig. 2:**
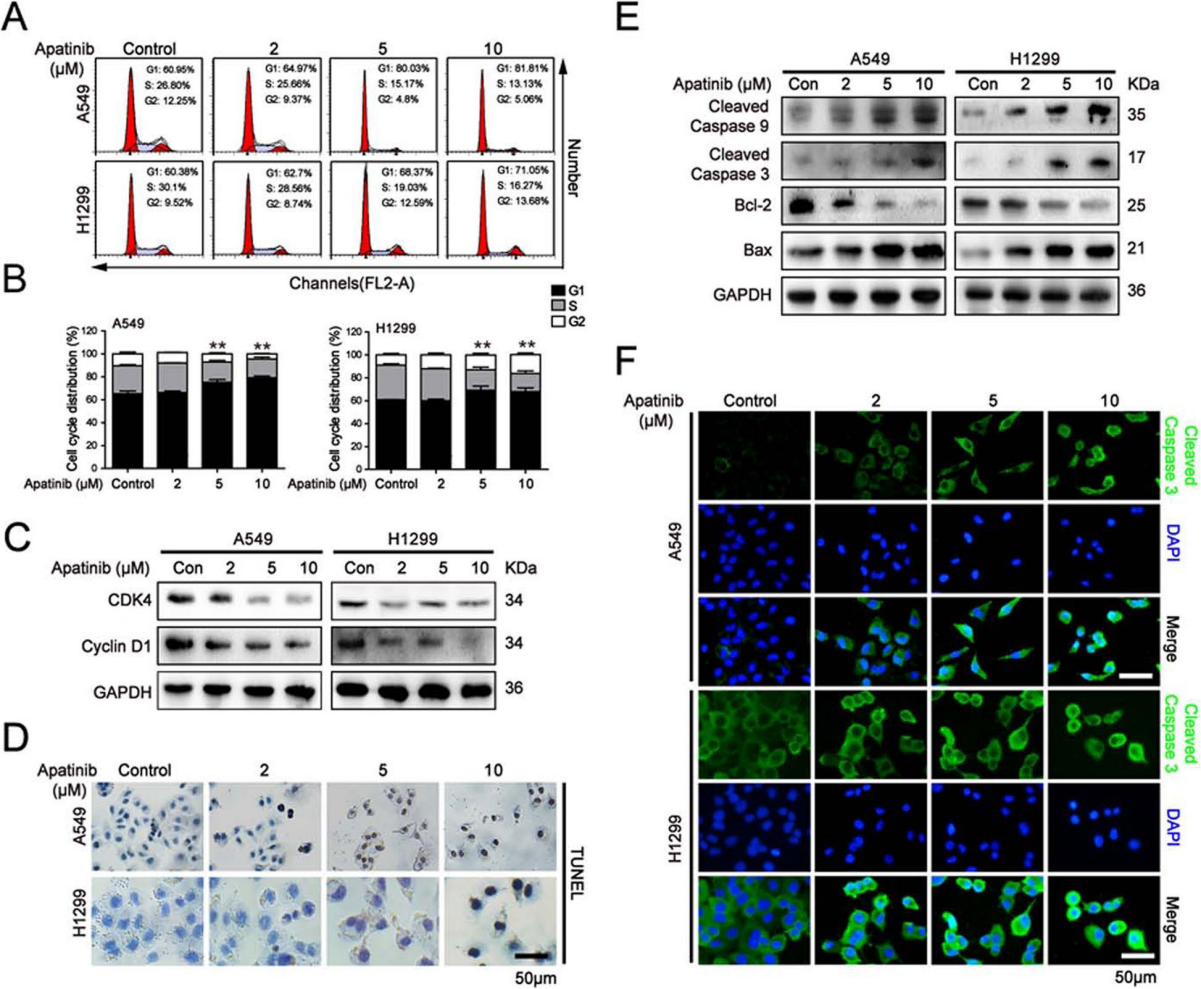
Apatinib induced G1 cell cycle arrest and apoptosis in NSCLC cells. A549 and H1299 cells were treated with indicated treatment for 48 h. **a** Flow cytometry assay of cell cycle distribution. **b** The quantitation of cell cycle distribution. The data are presented as mean ± SD (*n* = 3). **p < 0.01 vs. control. **c** Western blot analysis of G1 phase-related regulators CDK4 and Cyclin D1. **d** TUNEL staining of apoptosis in A549 and H1299 cells. Scar bar = 50 μm. **e** Western blot analysis of apoptosis-related proteins Cleaved Caspase 9, Cleaved Caspase 3, Bcl-2, and Bax expression. **f** Immunofluorescence staining of Cleaved Caspase 3 (Cleaved Caspase 3, green; DAPI, blue). Scar bar = 50 μm

**Fig. 4:**
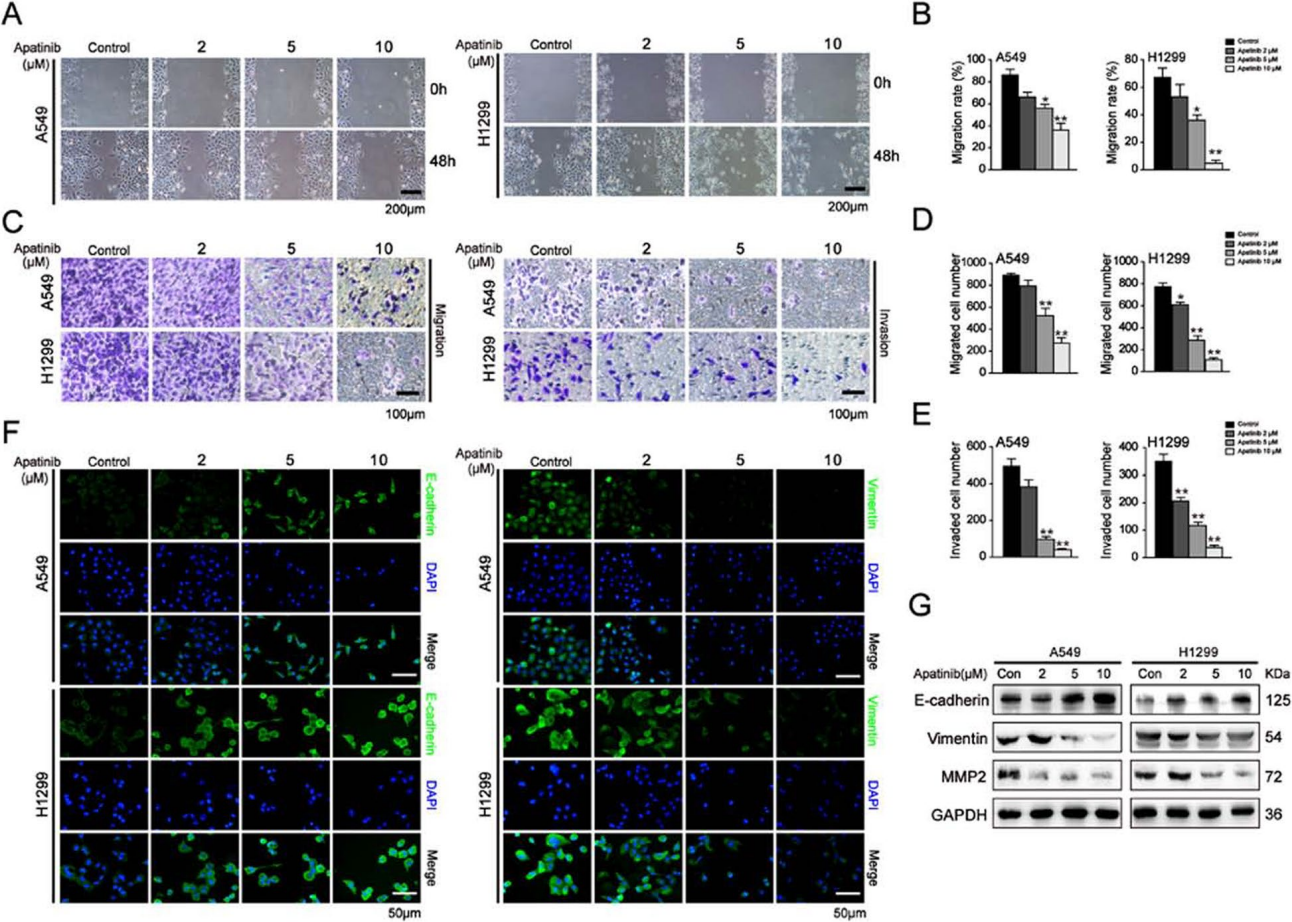
Apatinib suppressed the migration and invasion of NSCLC cells. A549 and H1299 cells were treated with apatinib for 48 h. **a** and **b** Wound healing assay was performed to evaluate the migration capacity. The migration rate was quantitated. Scar bar = 200 μm. The data are presented as mean ± SD (*n* = 3). *p < 0.05, **p < 0.01 vs. control. **c-e** Transwell assays without or with Matrigel were performed to evaluate the migration and invasion capacity. The numbers of migrated and invaded cells were quantitated. Scar bar = 100 μm. The data are presented as mean ± SD (*n* = 3). *p < 0.05, **p < 0.01 vs. control. **f** Immunofluorescence staining of E-cadherin and Vimentin (E-cadherin and Vimentin: green; DAPI: blue). Scar bar = 50 μm. **g** Western blot analysis of EMT-regulated proteins E-cadherin, Vimentin and MMP2

**Fig. 5:**
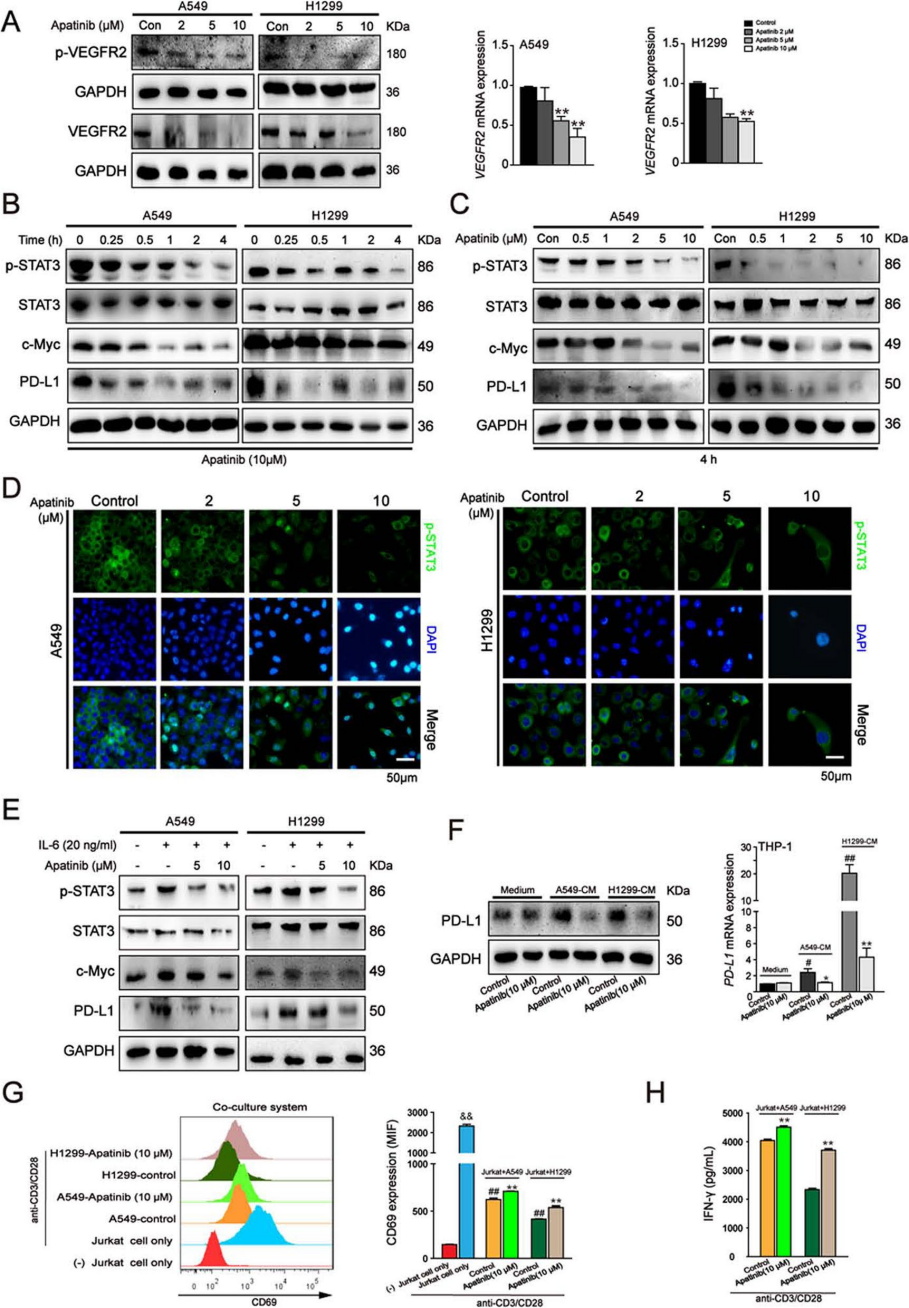
Apatinib downregulated VEGFR2/STAT3/PD-L1 pathway in NSCLC cells and reduced the immunosuppressive TME. **a** Western blot analysis of VEGFR2 and p-VEGFR2 (Tyr1175) expression and qRT-PCR analysis of VEGFR2 expression in A549 and H1299 cells after aptinib treatment for 48 h. The data are presented as mean ± SD (*n* = 3). **p < 0.01 vs. control. **b** and **c** Western blot analysis of p-STAT3, STAT3, c-Myc and PD-L1 expressions in A549 and H1299 cells after indicated treatment. **d** Immunofluorescence staining of p-STAT3 in A549 and H1299 cells after apatinib treatment for 4 h (p-STAT3: green; DAPI: blue). Scar bar = 50 μm. **e** Western blot analysis of p-STAT3, STAT3 and PD-L1 expressions in A549 and H1299 cells after apatinib treatment with or without pretreatment of IL-6 for 48 h. **f** Western blot and qRT-PCR analysis of PD-L1 expression in THP-1-derived macrophages with or without stimulation with CM from A549 and H1299 cells. The data are presented as mean ± SD (*n* = 3). *p <0.05, **p < 0.01 vs. A549 or H1299-CM control. ^#^*p* < 0.05, ^#^*p* < 0.01 vs. medium control. **g** and **h** Jurkat cells were activated by stimulation with anti CD3/CD28 antibodies and then co-cultured with non-treated or apatinib (10 μM) pretreated A549 or H1299 cells; the Jurkat cells were collected for CD69 detection by flow cytometry (**g**) and the co-culture medium was collected for IFN-γ secretion by ELISA assay (**h**). The data are presented as mean ± SD (*n* = 3). ^&&^ *p* < 0.01 vs (−) Jurkat cell only group. **p < 0.01 vs. A549 + Jurkat or H1299 + Jurkat control group. ^##^ *p* < 0.01 vs. Jurkat cells only stimulated with anti CD3/CD28 antibodies group
