# Supplementary material for: Apatinib triggers autophagic and apoptotic cell death via VEGFR2/STAT3/PD-L1 and ROS/Nrf2/p62 signaling in lung cancer
Source: J Exp Clin Cancer Res. 2021 Aug 24;40:266. doi: 10.1186/s13046-021-02069-4 (PMC8385858; doi:10.1186/s13046-021-02069-4)

Supplementary. Raw data for the blots included in each figure.

For Figure 2C.

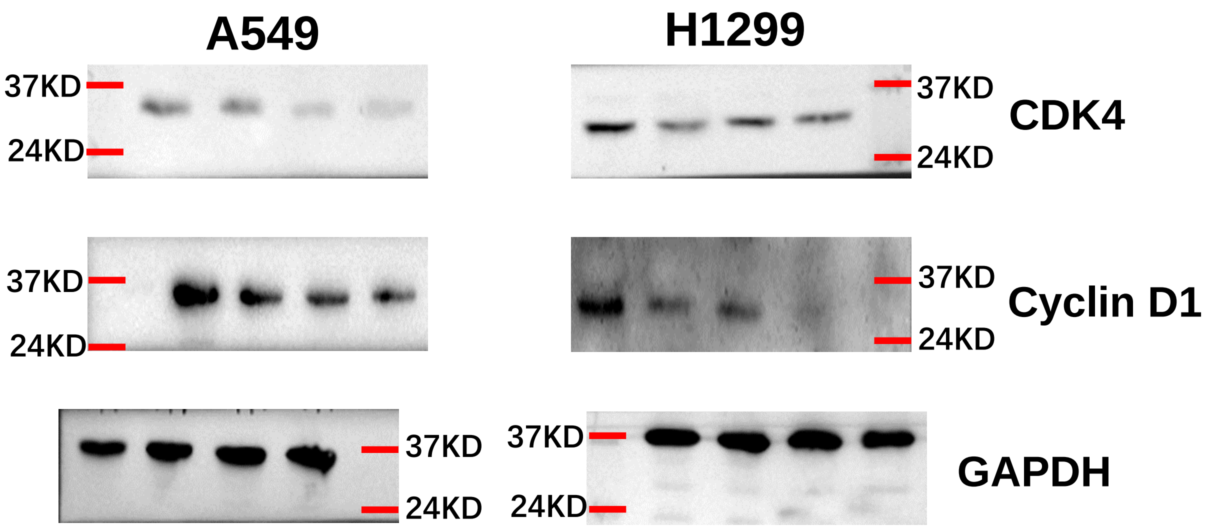

For Figure 2E.

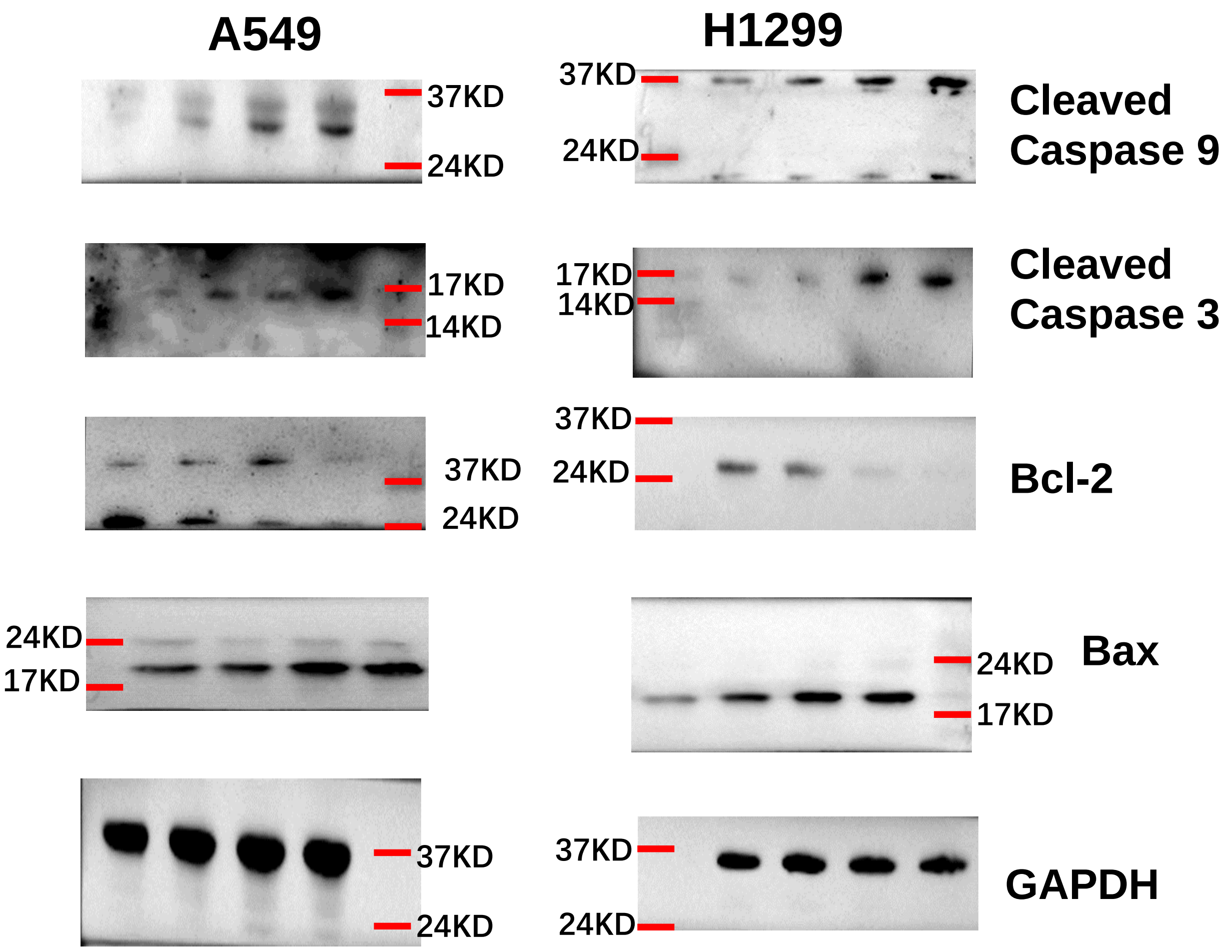

Supplementary. Raw data for the blots included in each figure.

For Figure 4G.

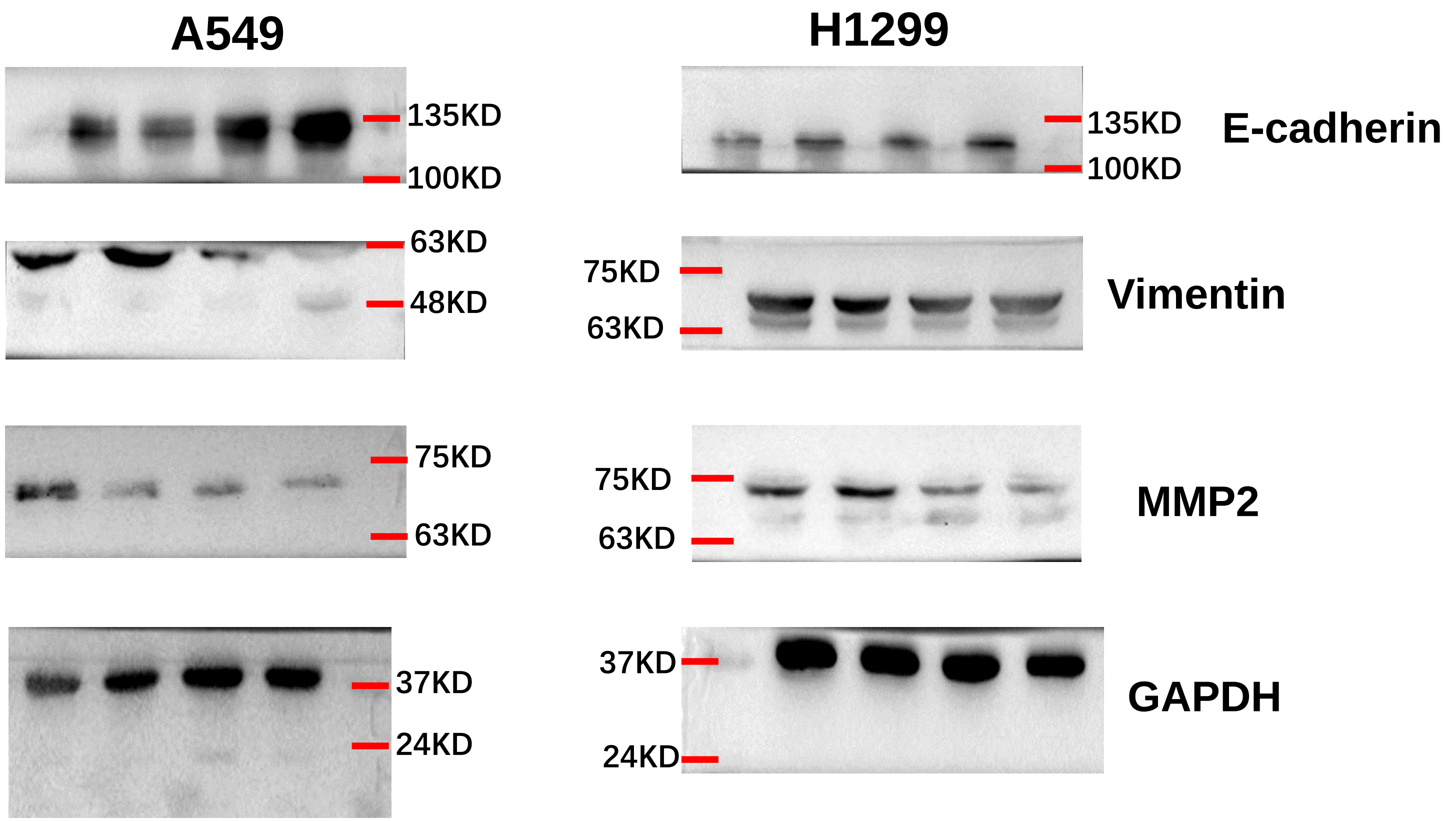

Supplementary. Raw data for the blots included in each figure.

For Figure 5A.

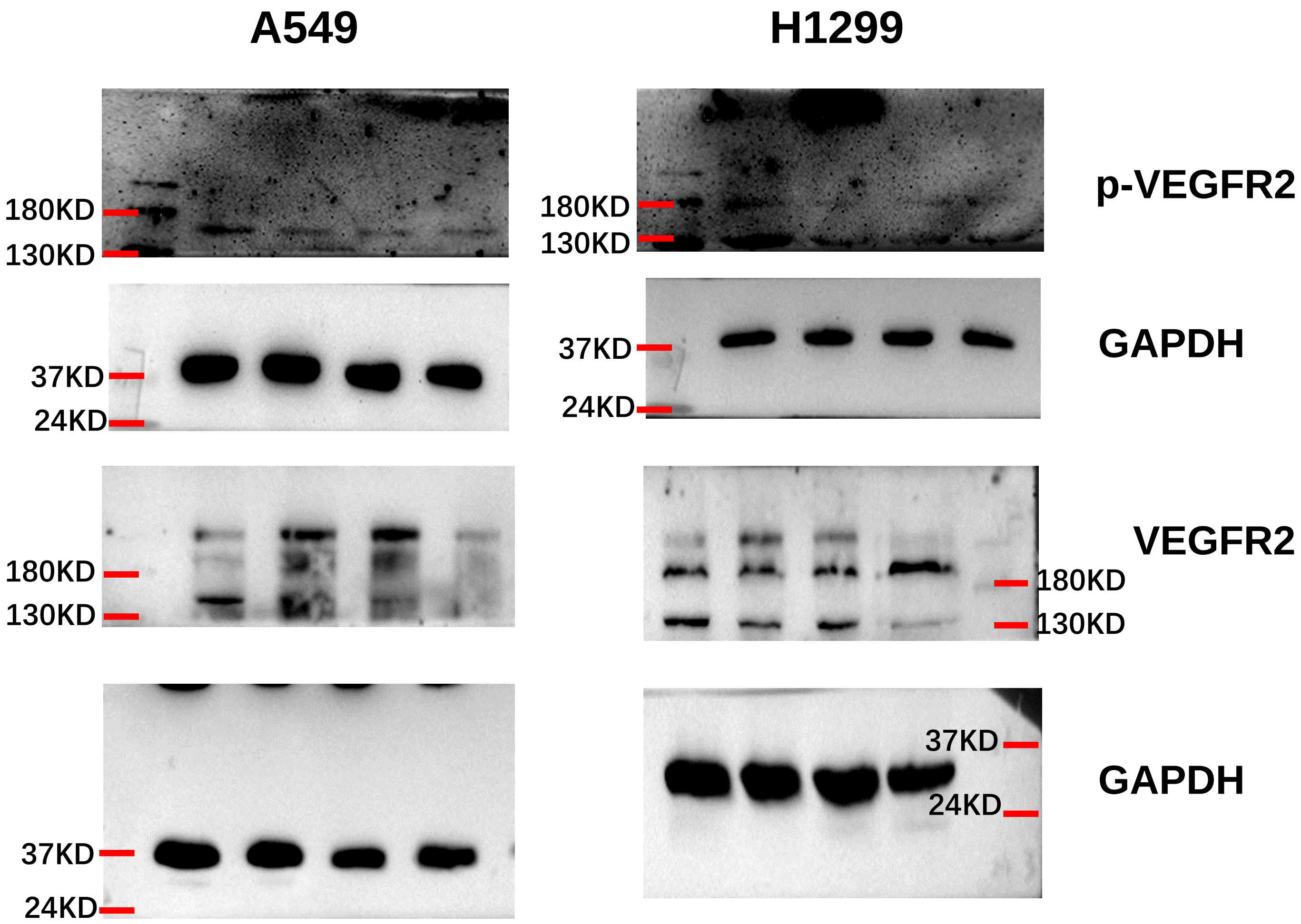

Supplementary. Raw data for the blots included in each figure.

For Figure 5B.

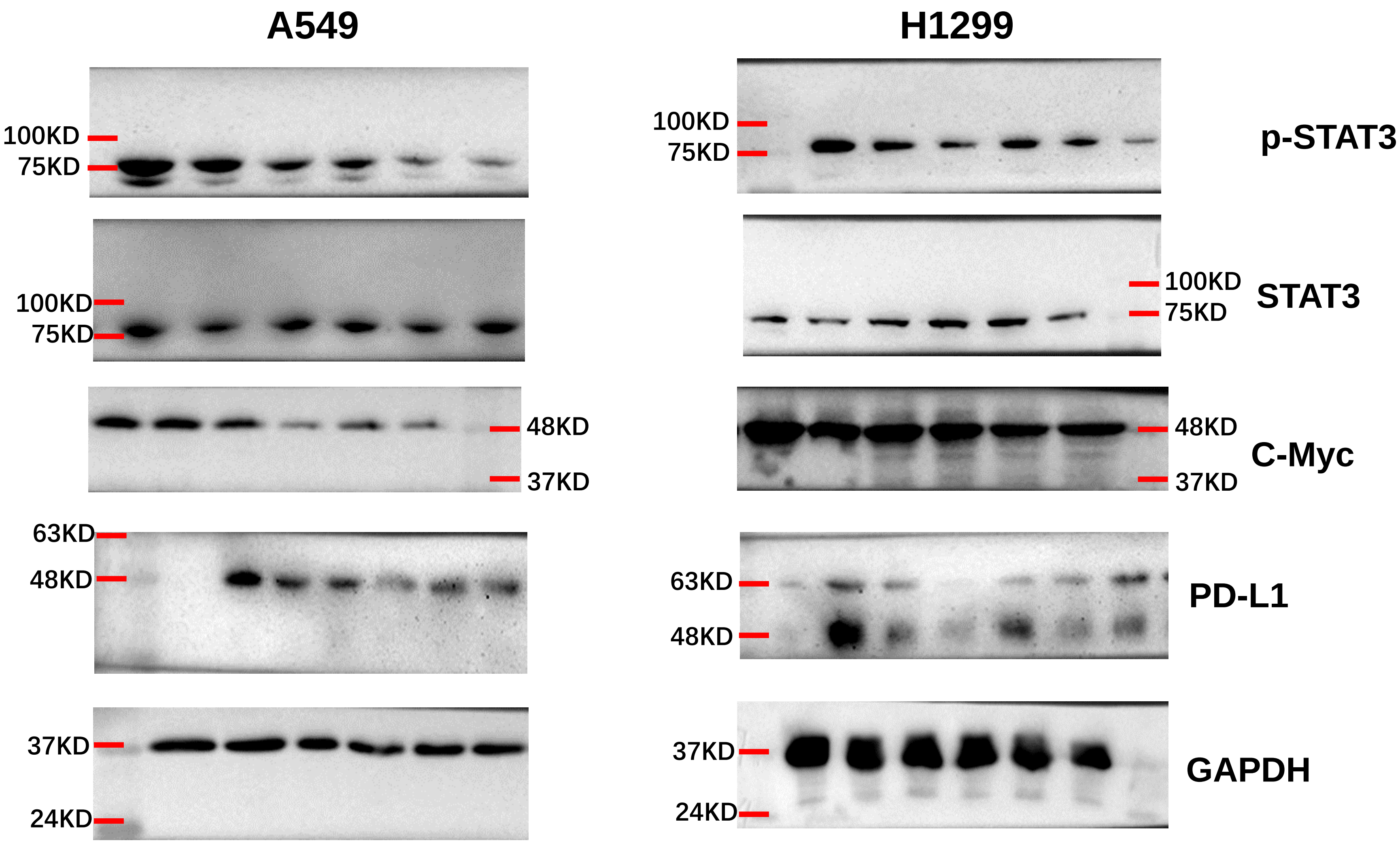

Supplementary. Raw data for the blots included in each figure.

For Figure 5C.

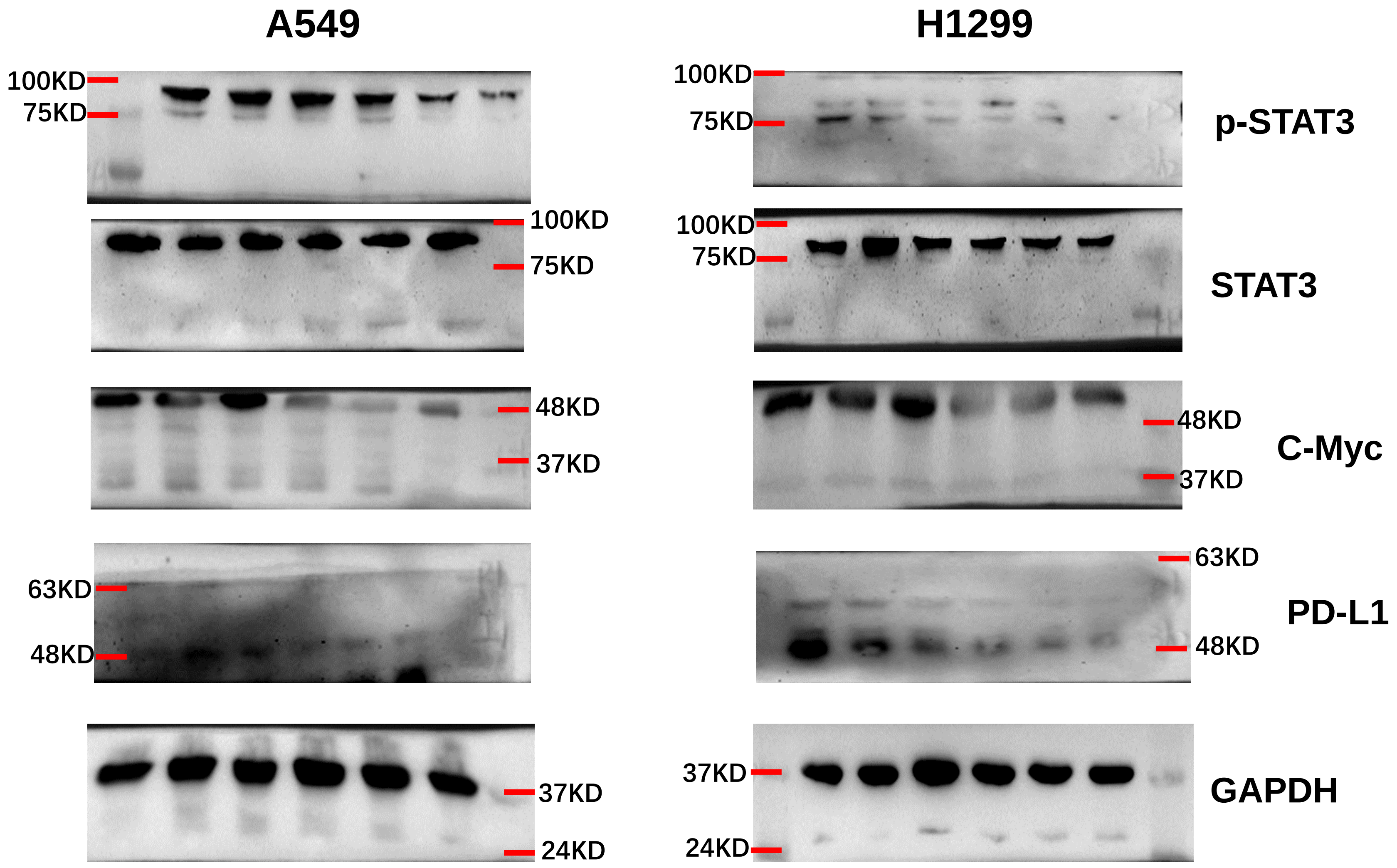

Supplementary. Raw data for the blots included in each figure.

For Figure 5E.

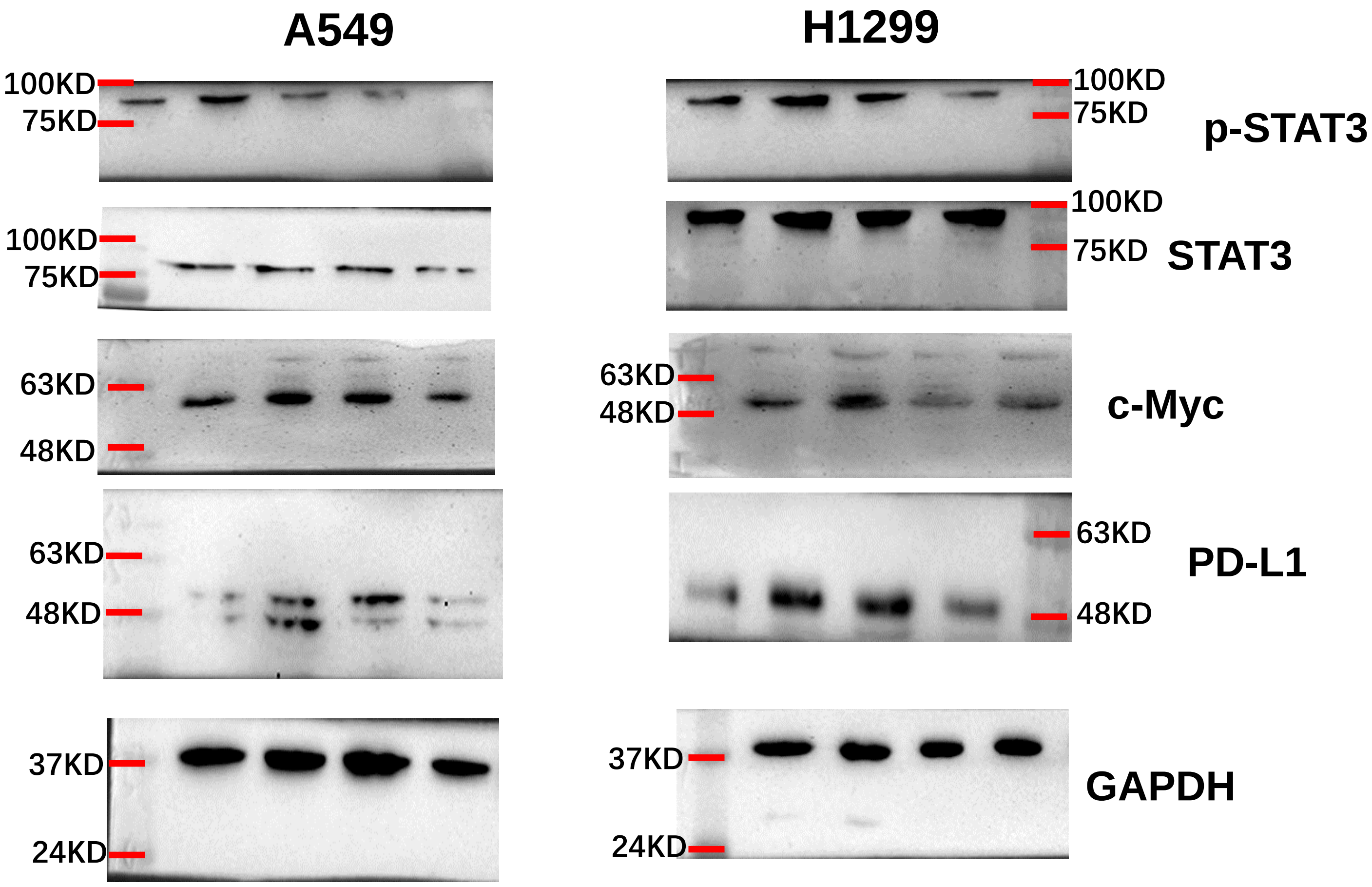

For Figure 5F.

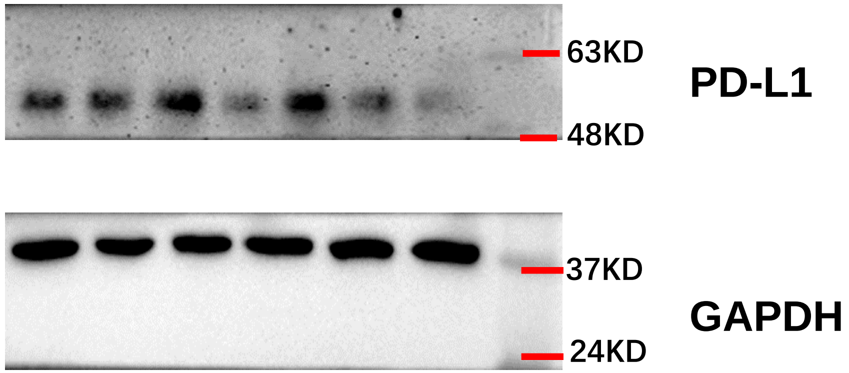

Supplementary. Raw data for the blots included in each figure.

For Figure 6B.

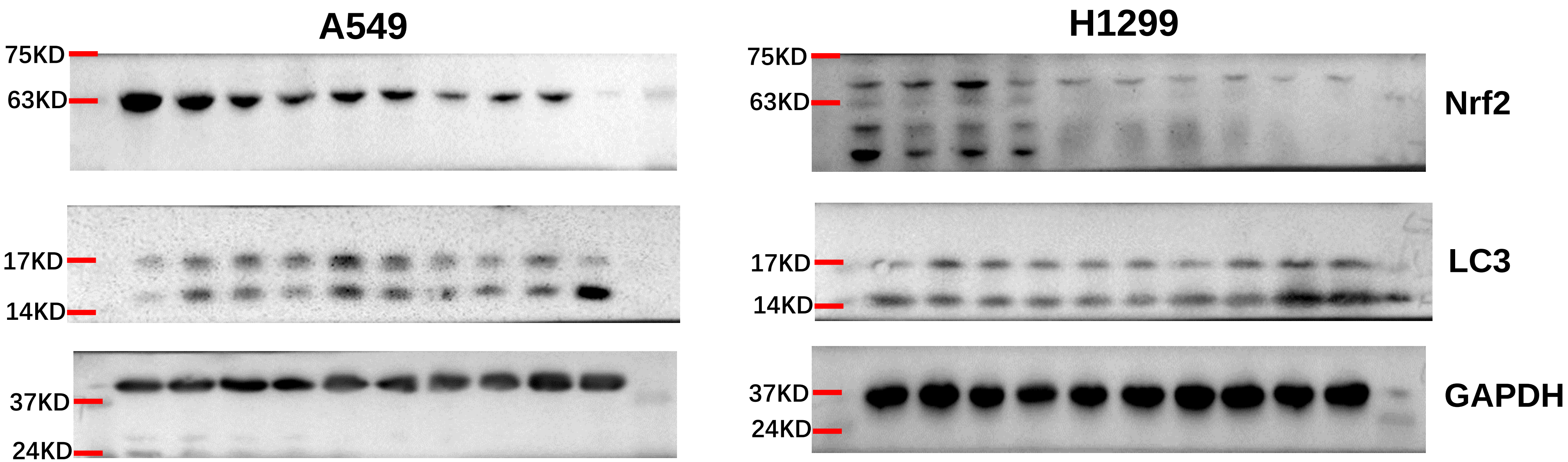

Supplementary. Raw data for the blots included in each figure.

For Figure 6C.

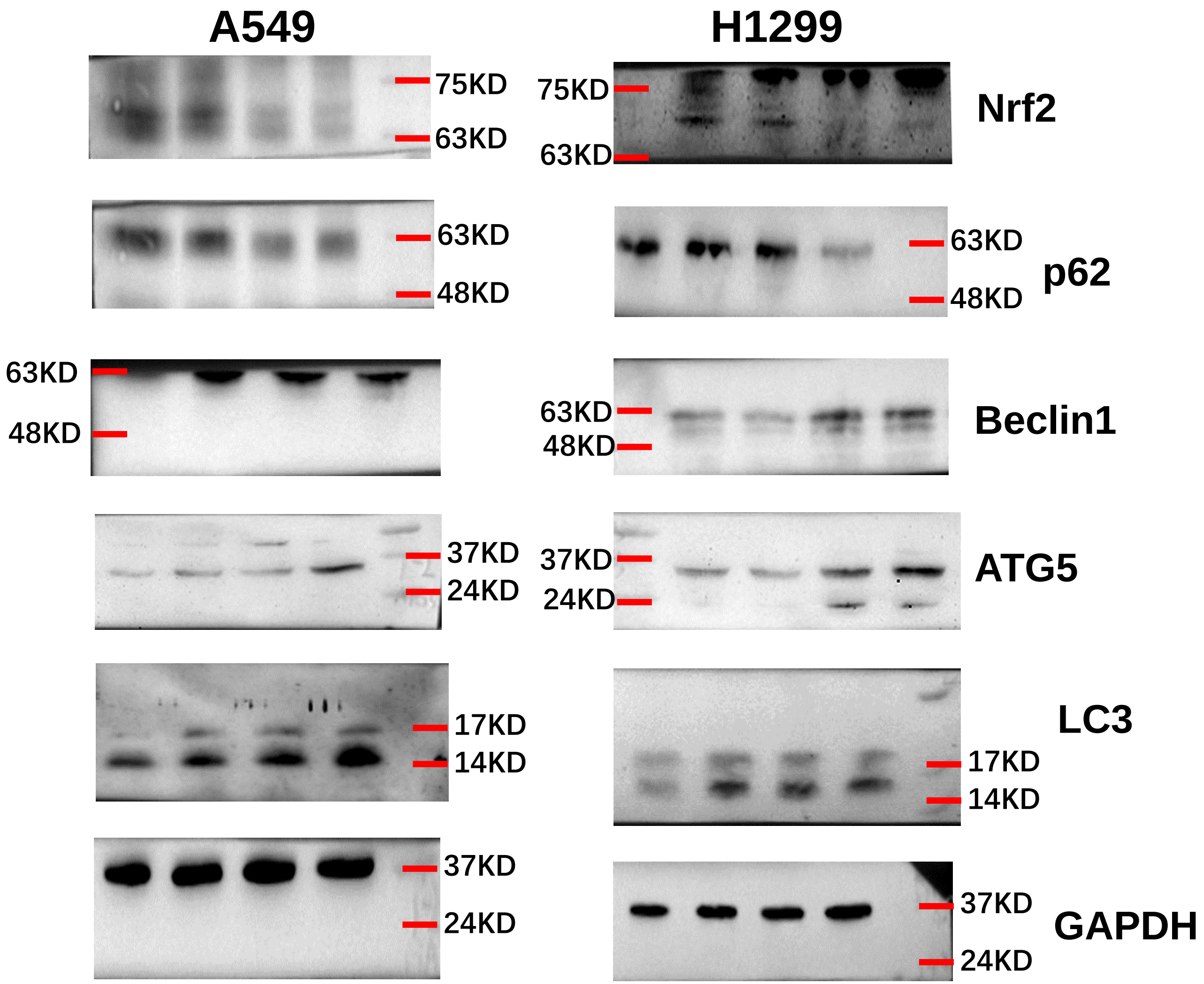

For Figure 6G.

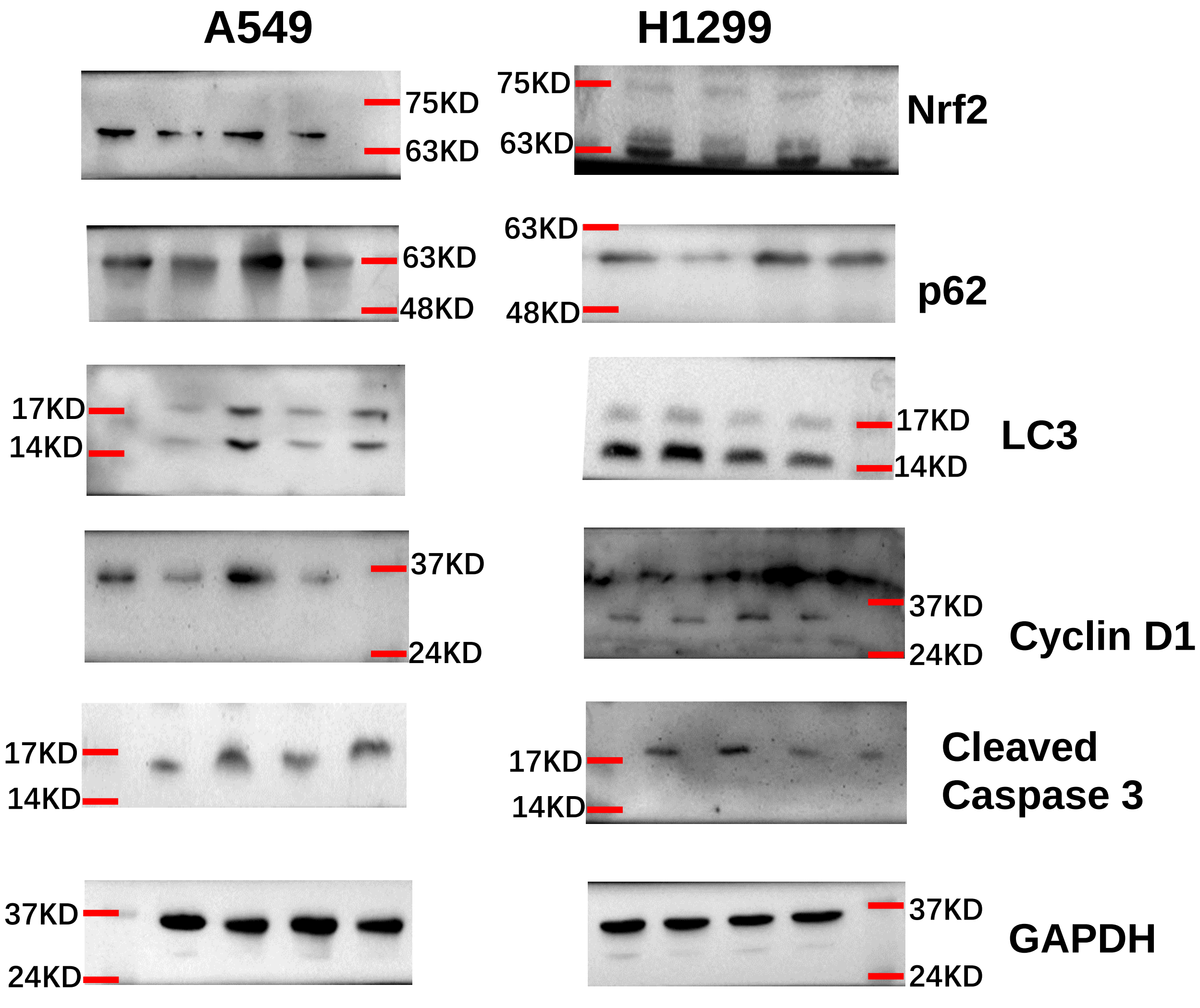

Supplementary. Raw data for the blots included in each figure.

For Figure 6B.

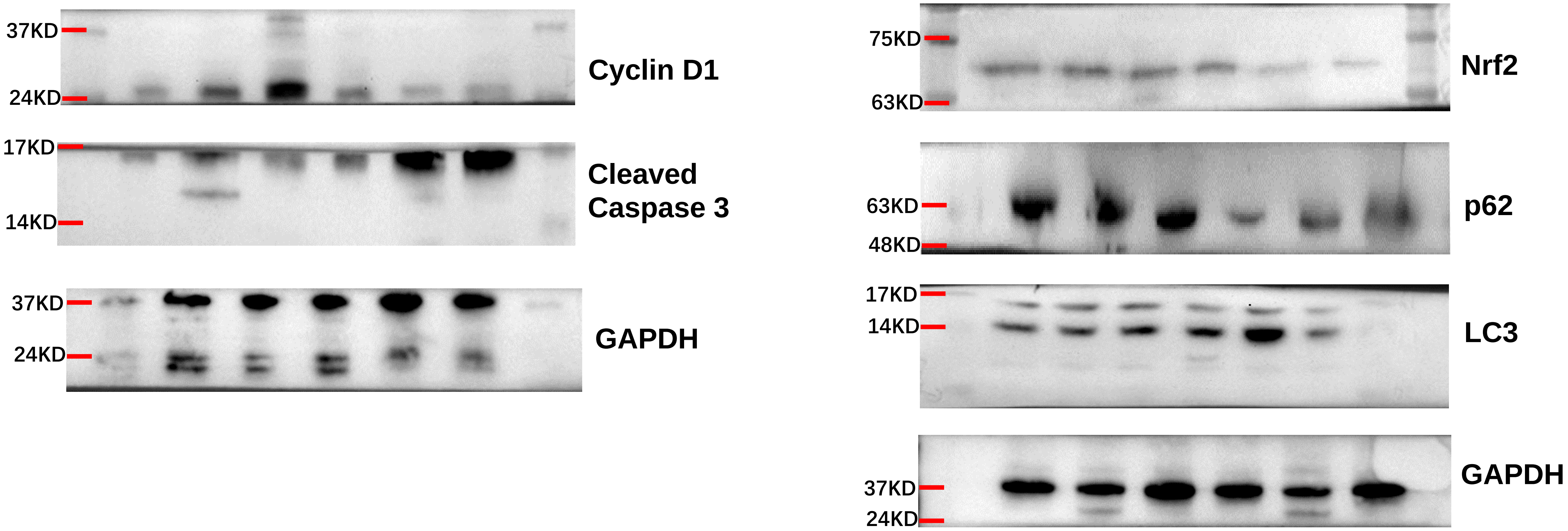

Supplement: Supplementary file 1 — Additional file 1:Supplementary. Raw data for the blots. [file 13046_2021_2069_MOESM1_ESM.pdf]
